# Autism, intelligence, language, and adaptive behavior, disentangling a complex relationship

**DOI:** 10.3389/fpsyt.2024.1411783

**Published:** 2024-11-06

**Authors:** Chiara Failla, Ileana Scarcella, Noemi Vetrano, Serena Previti, Rita Mangano Mangano, Gennaro Tartarisco, David Vagni, Giovanni Pioggia, Flavia Marino

**Affiliations:** ^1^ Institute for Biomedical Research and Innovation (IRIB), National Research Council of Italy (CNR), Messina, Italy; ^2^ Classical Linguistic Studies and Education Department, Kore University of Enna, Enna, Italy; ^3^ Faculty of Psychology, International Telematic University Uninettuno, Roma, Italy; ^4^ Department of Cognitive, Psychological Science and Cultural Studies, University of Messina, Messina, Italy

**Keywords:** adaptive behavior, adaptive functioning, autism, cognitive development, intelligence quotient, ICD-11 classification, functional language, parental age

## Abstract

**Introduction:**

Autism Spectrum Disorder (ASD) is a complex neurodevelopmental disorder characterized by a range of intellectual and language abilities. Its heterogeneity is acknowledged in modern diagnostics, complicating research and necessitating precision medicine and a multidimensional approach for individualized treatment and accurate assessment. Intellectual and language functioning influence adaptive skills and symptomatology. Thus, assessing adaptive functioning in a multidimensional and multi-informant manner is crucial, highlighting the importance of comprehensive evaluations. This study explores the interplay between autistic traits, demographic variables, IQ, adaptive functioning, and the applicability of ICD-11 classifications.

**Methods:**

We analyzed data from the initial global evaluation of 60 diverse autistic children (aged 35 to 120 months; IQ range 16 to 118). Parent-reports using the Vineland Adaptive Behavior Scale (VABS-II) were compared with standardized assessments from the PsychoEducational Profile (PEP-3). Children’s intellectual levels were assessed using Griffiths Scales of Child Development (Griffiths III) and the Autism Diagnostic Observation Schedule (ADOS-2) was used for autistic traits. They were further classified according to the ICD-11 diagnostic system, functional language, and intellectual functioning levels. Correlations among variables, group comparisons, and multivariate analyses were performed.

**Results:**

The analysis indicates a linear effect of IQ on all adaptive scales and the impact of autistic traits on directly measured adaptive functioning. A factorial effect was observed due to changes concerning specific age, intellectual, and linguistic levels, which do not completely align with ICD-11 categorization. Additionally, a negative correlation between intelligence and measured autistic traits was found. Parental age, education level, and age at childbirth were also found to affect various adaptive scales.

**Discussion:**

The study questions the ICD-11’s proposed distinctions in IQ and language functioning for ASD, advocating for more refined categorization and developmental considerations. It underscores the intricate relationship between autistic traits, IQ, and communication skills, casting doubt on the precision of diagnostic tools across the spectrum. Parental reports and direct assessments are essential for comprehensive evaluation, with parental education and age influencing children’s behaviors and skills. The study calls for a nuanced approach to ASD assessment, integrating various metrics and sources of information for a detailed phenotyping necessary for personalized interventions and biological research.

## Introduction

Autism Spectrum Disorder (ASD) is a complex neurodevelopmental condition characterized by persistent deficits in social communication and restricted repetitive behaviors. ASD comprises diverse etiologies, involving a variety of genetic and environmental factors (1). Genetic heterogeneity has been widely demonstrated through genetic association studies and research on rare and common genetic mutations, confirming the presence of multiple genetic variants associated with autism (2).

In the past, autism was often associated, if not conflated, with intellectual disability and language disorders. Early research and clinical observations led to the perception that individuals with autism universally exhibited cognitive impairments and significant language deficits. This prevailing view contributed to the stigmatization of autistic individuals and limited our understanding of the condition. However, contemporary insights have shifted this paradigm. We now recognize that autism is highly heterogeneous (3), with many individuals demonstrating average or above-average intelligence, and age-appropriate language skills (4).

In a large USA sample of 8-year-old children with ASD who underwent cognitive assessments, 37.9% were identified as having intellectual disability, 23.5% fell within the borderline range (IQ 71–85), and 38.6% were classified as having average or above-average intelligence (IQ >85) (4).

ASD has undergone significant redefinition in recent diagnostic classification systems, notably the DSM-5-TR (5) and ICD-11 (6). Both frameworks now employ the umbrella term “Autism Spectrum Disorder” to encompass a diverse array of conditions previously categorized separately. While this harmonization facilitates clinical communication and streamlines diagnostic criteria, it introduces challenges related to the inherent heterogeneity within ASD. The heterogeneity inherent in the ICD-11 and DSM-5-TR conceptualization threatens research reproducibility, and studies may struggle to replicate findings due to the broad spectrum captured. To advance our understanding of etiology and biological pathways, we must move beyond the current paradigm; because precision medicine, predicated on individualized treatment strategies, necessitates a more granular description of symptoms. Therefore, core ASD subtypes or endophenotypes should emerge, allowing targeted investigations. Within this mosaic of ASD, profound autism (minimally or absent functional language and IQ < 50) and giftedness (presence of functional language and IQ > 130) represent two poles. Profound autism, characterized by severe impairments in communication, intellectual development, and daily functioning, demands very intensive support (7). Conversely, gifted individuals with ASD exhibit exceptional cognitive abilities, necessitating tailored enrichment and specific psychoeducational intervention (8). Between these two poles lies a vast array of functioning levels that must be addressed specifically. Moreover, ASD’s profile is multidimensional, encompassing frequent co-occurring conditions (9), discrepant verbal and non-verbal abilities, islets of ability, diverse biological pathways, and distinct educational and therapeutic needs. This complexity underscores the importance of assessing not only IQ but also various other clinical variables to differentiate between different ASD phenotypes.

In current diagnostic systems (DSM-5 and ICD-11), language deficits are no longer part of the core diagnostic criteria for ASD. However, both systems require the specification of any accompanying language impairment or intellectual disability, or intellectual developmental disorder (IDD) according to ICD-11. At the same time, it is required to specify any other co-occurring conditions, such as anxiety disorders or attention deficit and hyperactivity disorder, or the presence of known medical and genetic conditions. The presence of co-occurring conditions can greatly influence adaptive functioning and well-being and can also affect how ASD core symptoms appear and change over time (10). To fully understand an autistic person, both core symptoms and other challenges should be considered. Furthermore, other factors, such as daily living skills, support needs, and environmental resources, should also be considered in evaluating disability in autistic individuals. A multidimensional approach to autism, could offer a more comprehensive system for classifying impairment (11). However, there is no formal way to measure the combined effect of these different aspects of autism on a person’s life.

Many studies on the prevalence of IDD in autistic children use only cognitive tests, despite the diagnostic criteria requiring adaptive skills assessment (4, 12–15). Intelligence quotient scores alone are not sufficient to measure IDD, as recent studies have shown (16). A more comprehensive approach is needed, which includes adaptive functioning (AF) - a set of age-appropriate skills for communication, social interaction, and everyday life (17). AF reflects the real-life challenges faced by individuals with IDD and should be considered in any accurate assessment. A perspective that recognizes impairments, capabilities, co-occurring conditions, and environmental factors would help identify subgroups of individuals and assess their individual needs and strengths.

Furthermore, in many clinical settings, the language skills of children with ASD are not always thoroughly assessed, and subtle distinctions between different linguistic abilities are often missed. Studies have reported diverse language profiles in ASD, with some children having intact structural language skills and others displaying language impairments like those seen in Developmental Language Disorder (DLD) (18). These deficits cause significant limitations in communication ability. While the ICD-11 specifies that language deficits in DLD are “not better accounted for by Autism Spectrum Disorder,” it also acknowledges that an additional DLD diagnosis should be assigned when there are “additional specific impairments in semantic, syntactic, and phonological development.” In ICD-11, three classes of Functional Language (FL) are present: Mild or No Impairment in Functional Language (MNIFL), intact structural language skills comparable to neurotypical language; Impaired Functional Language (IFL), impaired structural language skills (phonology and/or morphosyntax); Absent Functional Language (AFL), minimal verbal abilities, limited expression with a restricted set of words or absence of spoken language.

Therefore, ICD-11 classifies five different subcategories in ASD. They derive from all possible combinations of the previously described functional language and intellectual abilities. To diagnose IDD in ASD, both intellectual impairment and adaptive functioning deficits must be present, and the assessment should be focused on daily living skills because social and communication difficulties are already a component of ASD criteria.

The ICD-11 classifies ASD based on IDD and language skills, as shown in **Table 1**.

**Table 1:** ICD-11 autism spectrum disorder classification.

Intellectual development disorder (IDD)	Language skills		
	mild or no impairment	impaired	absent
without IDD	6A02.0	6A02.2	–
with IDD	6A02.1	6A02.3	6A02.5

Two other residual categories were added, 6A02.Y Other specified autism spectrum disorder, and 6A02.Z Autism spectrum disorder, unspecified.

Cognitive functioning influences adaptive functioning in ASD, but not in a straightforward way (19). Lower IQ is associated with poorer adaptive behaviors in ASD with IDD, as Kanne et al. (20) suggested, but ASD without IDD may also show lower adaptive skills than expected from their IQ (21). Vineland Adaptive Behavior Scales (VABS) scores are significantly below IQ scores in children without IDD (22–25). This may be due to increased symptoms such as attention, hyperactivity, and emotional problems, which impair adaptive abilities in children without IDD. However, Tillmann et al. (19) did not find this link and instead related it to the social and communication styles of autistic people. They also did not find a link with the ADOS-2 score and only used self/other report of autistic traits, which may introduce confounding factors. Parent reports are a common source of information about children’s development, including autistic children. However, few studies have examined their predictive value for directly assessed measures of adaptive functioning in ASD. Previous studies have shown that parental estimates vary depending on the child’s age, cognitive ability, autistic traits, and adaptive skills. For instance, parents tend to overestimate IQ when it is low or adaptive skills are high (26). Nevertheless, parent reports of the child’s language and fine motor skills are usually consistent with direct testing, regardless of the diagnosis (ASD vs. other). When there is a discrepancy, parents are more likely to report a skill as present than observed on direct testing (27).

The purpose of this study is to investigate the relationship among autistic traits, adaptive functioning, IQ, and demographic variables. We are interested in the association between adaptive functioning as reported by parents and as assessed by professionals in autistic children, and how it is related to children’s autistic traits and IQ. We are also interested in how the parent-reported adaptive functioning measured by the VABS-II test affects the child’s observed adaptive skills measured with PEP-3, and how it is influenced by the child’s clinical presentation, such as the presence or absence of IDD, language delay, and overall autistic traits, we expect to find both linear and categorical effect.

Therefore, we have four main objectives:

1. To identify which clinical and demographic variables have the most impact on the child’s outcomes.
2. To understand if differences in adaptive behaviors vary linearly with IQ, functional language, and autistic traits or if there are also categorical differences among different diagnostic groups.
3. To explore how parents perceive their autistic children’s behavior, social and communication skills, and the relationship among demographic and clinical variables.
4. To evaluate if the ICD-11 classification gives an adequate representation of the variability within the autistic spectrum.

To achieve these objectives, we will collect parent-reported data using the VABS-II test, and compare it with a standardized assessment, the PEP-3 test, administered by professionals. We will also measure the child’s intellectual level using intelligence tests and ADOS-2 scores and classify the child according to the ICD-11 diagnostic system.

## Methods

### Study sample

We recruited 60 autistic children from the National Research Council (CNR) main office at IRIB in Messina. We assembled our study sample from children participating in various experiments within our project. Specifically, we included all children who underwent their initial assessment during the check-in process. This sample represents the baseline data for our investigation.

The sample age ranged from 35 to 120 months, with a mean of 68.7 months and a median of 65 months. The age distribution was slightly skewed to the right, with a standard deviation of 22.6 months. We used different instruments to assess cognitive functioning, depending on the age and ability of the children. We estimated IQ from developmental quotients using the Griffiths Scales for children under 6 or with low cognitive abilities. We used the Leiter-3 test for non-verbal children and the WISC-IV for children who could complete a more comprehensive assessment. This way, we adapted the cognitive evaluation to each child’s needs and capabilities. The IQ scores varied from 15.7 to 118, with a mean of 63.4 and a median of 65. The standard deviation of IQ was 24.7, indicating high variability in line with the one reported in the literature (28). The sample was diverse in both age and IQ, representing a heterogeneous group of participants. The ADOS-2 calibrated severity score (ADOS-2-CS) in our sample had a mean of 7.18 and a median of 7, with a standard deviation of 1.41. This showed moderate variability in the sample, assessed with module 1 and module 2.

### Measures

#### ADOS-2 autism diagnostic observation schedule-second edition

The ADOS-2 (29) is a semi-structured diagnostic child observation that captures social effect and stereotyped behavior and restricted interests in four alternative modules: 1—preverbal/single words, 2—phrase speech/non-fluent, 3—fluent speech (child/adolescent), and 4—fluent speech (adolescent/adult). Each module is specifically crafted to accommodate the communication abilities of the individual being assessed, ranging from preverbal stages to fluent speech in various age groups. The ADOS is widely used in clinical settings and research to aid in the diagnosis of ASD by providing a structured and standardized means of observing and evaluating social communication and behavioral patterns.

#### Cognitive functioning

The Griffiths Scales of Child Development (30) is a comprehensive developmental assessment tool designed for children from birth to 6 years of age. This assessment evaluates a broad range of developmental domains, including locomotor, personal-social, hearing and speech, eye and hand coordination, and performance. It provides a detailed examination of a child’s cognitive and motor development, offering insights into their strengths and potential areas for support. Griffiths Scales has a high to very high concurrent validity with measures of intelligence (31).

The Leiter International Performance Scale (third edition) (32), commonly known as the Leiter test, is a nonverbal intelligence test designed to assess cognitive abilities without relying on verbal language skills. The Leiter test places a particular emphasis on assessing nonverbal reasoning, visual-spatial processing, and working memory. It consists of a series of tasks that require the examinee to solve problems using abstract symbols and visual stimuli, making it suitable for individuals with limited verbal abilities or those from diverse linguistic backgrounds. The Leiter test is often employed in cases where traditional verbal assessments may not be applicable or effective, providing an alternative means to evaluate cognitive functioning.

The Wechsler Intelligence Scale for Children, Fourth Edition (WISC-IV), is a widely used intelligence test designed for assessing the cognitive abilities of children aged 6 to 16 years and 11 months (33). This comprehensive test is structured into various subtests, covering areas such as verbal comprehension, perceptual reasoning, working memory, and processing speed. The WISC-IV aims to provide a detailed profile of a child’s cognitive strengths and weaknesses, offering valuable insights for educational and clinical purposes.

Our sample of 60 children underwent cognitive assessment with a distribution as follows: 78% (n=47) of the children were assessed using the Griffiths scale, 14% (n=8) underwent evaluation through the Leiter 3, and 8% (n=5) underwent assessment using the WISC-IV.

#### PEP-3 Psychoeducational Profile-3

The PEP-3 (34) is a scale used for functional assessment in children with autism between the ages of two and seven years. This scale identifies the strengths and weaknesses in the main areas of a child’s behavioral development. It is divided into two sections. Before the assessment of the child, the caregivers complete a questionnaire. This report is divided into 3 sub-tests that investigate problem behaviors (10 items), personal autonomy (13 items), and adaptive behaviors (15 items). The test score is quantified as “0,” “1” and “2”. This is followed by the performance section, filled in by the examiner during direct observation of the child. This section consists of 10 subtests, 6 measure developmental skills, and 4 measure maladaptive behaviors. The subtest of developmental skills is subdivided into verbal/preverbal cognitive subtest (CVP) (34 items); expressive language subtest (LE) (25 items); receptive language subtest (19 items); fine motor subtest (MF) (20 items); global motor subtest (MG) (15 items); visual-motor imitation subtest (IVM) (10 items) (Schopler et al., 2006). Instead, the subtests of maladaptive behaviors, renamed by us characteristic behaviors (CB), are Emotional Expression (EE) (11 items); Social Reciprocity (RS) (12 items); Characteristic Motor Behaviors (CMB) (15 items); Characteristic Verbal Behaviors (CVB) (11 items). The combination of the developmental subtests and maladaptive behaviors constitutes 3 composite scores of Communication (PEP-3-C), Motor Skills (PEP-3-M), and Characteristic Behaviors (PEP-3-CB). It is important to note that higher scores on PEP-3-CB mean less maladaptive behaviors.

#### VABS-II Vineland Adaptive Behavior Scales

Parents have completed the Vineland Adaptive Behavior Scales-2 (VABS-2) Parent/Caregiver Rating Form developed by Sparrow et al. (35). The VABS-2 evaluates adaptive behavior across domains such as communication, daily living skills, and socialization. Standard scores are provided for communication (VABS-2-C), daily living skills (VABS-2-DL), and socialization (VABS-2-S), along with an adaptive behavior composite. The standard scores for VABS-2 range from 20 to 160, with a mean of 100 and a standard deviation of 15. Internal consistency reliabilities for domain and composite scores on the VABS-2 range from 0.80 to 0.97, and test-retest reliability correlations for 7-12-year-old children range from 0.75 to 0.93 (Sparrow et al., 2005).

### Ethical clearance

The study was conducted according to the guidelines of the Declaration of Helsinki and approved by the Committee of the Research Ethics and Bioethics Committee (http://www.cnr.it/ethichs, accessed on 17 December 2021) of the National Research Council of Italy (CNR) (Prot. No. CNR-AMMCEN 54444/2018 01/08/2018) and by the Ethics Committee Palermo 1 (http://www.policlinico.pa.it/, accessed on 17 December 2021) of Azienda Ospedaliera Universitaria Policlinico Paolo Giaccone Palermo (report No. 10/2020 - 25/11/2020). All participants were given verbal and written information about the study procedures and that they could withdraw from study participation at any time without further explanation. The parents involved in the study have formally signed an informed consent before the assessments. By signing the consent, they have agreed to the administration of cognitive and behavioral tests on their child. Additionally, they have accepted the responsibility to complete the questionnaire on their child’s adaptive functioning.

### Statistical analysis

The study is exploratory, so we will employ various methods and analyses to dissect the variable space. We will not apply complex modeling due to the small and convenient nature of the sample size. PEP-3 communication and motor scales have both ASD specific and general population norms, while maladaptive/characteristic behavior scale has only an ASD specific norm. Furthermore, values are reported as percentile ranks. Given our interest in subgrouping ASD participants, we used ASD specific norms and to enhance data normality and consistency with other variables, we transformed PEP-3 original scales to reach a standardization with mean (M) of 100 and a standard deviation (SD) of 15 (36).

After classifying participants into different IQ and functional language levels according to ICD-11 classification, we conducted a group-wise descriptive analysis using the Independent-Samples Kruskal-Wallis Test, with Bonferroni correction for pairwise comparisons. Bivariate correlation analyses were performed using the Pearson correlation coefficient. In **Supplementary Materials**, we also reported results using the Spearman correlation coefficient.

For parametric analyses (ANOVA, ANCOVA, MANOVA, MANCOVA), normality and homogeneity were not violated unless explicitly stated otherwise in the results section. Normality was assessed using the Shapiro–Wilk test, and homogeneity was assessed using Box’s M test. We used the Modified Breusch-Pagan Test to assess heteroskedasticity, and Levene’s test was employed for *post hoc* comparisons.

Factor analysis was performed using principal component analysis with eigenvalues greater than 1, followed by varimax rotation with Kaiser normalization.

Since the study was exploratory and based on a rolling sample, we did not conduct a sensitivity analysis. The **Supplementary Materials** include the observed power for all the analyses and complete statistics related to assumptions and alternative models. Raw data was not reported in the **Supplementary Materials** or repositories because, given the large number of variables and the nature of the sample, it would render the participants identifiable and therefore violate their privacy. A curated version without demographic information is included in **Supplementary Data Sheet 12.**

For all our analyses, we employed a two-sided test with an alpha level of 0.05. In the case of parametric analyses, we adjusted the significance level using the Šidák correction. It’s worth noting that even though we planned to conduct multiple analyses (with corrections applied only within each analysis, not across the entire study), we anticipated a strong correlation among variables. Highly correlated variables tend to move together, reducing the likelihood of false positive results (Type I errors). Consequently, the effective degrees of freedom are diminished. To ensure the robustness of our findings, we employed various methods to assess relationships among variables, guarding against undue influence from specific statistical techniques or assumptions. Throughout our analyses, we gradually introduced new sets of correlates to show the impact of their inclusion and to use them as “previous” hypotheses for more complex analyses. Unless explicitly specified otherwise, we assumed that directionality would remain consistent. Therefore, we reported p-values between 0.05 and 0.10 if the previous test yielded a value less than 0.05. Given the exploratory nature of our study, we were cautious about avoiding Type II errors — missed opportunities. Balancing this risk with the need for a thorough exploration of relationships was crucial.

We utilized SPSS software (v. 29, IBM Corporation, Armonk, NY, USA) for statistical analyses.

## Results

The full statistical analysis and detailed numerical results are provided in the “Full Results” file in the **Supplementary Materials**. This file includes all correlations, model assumptions, and complete data sets for each analysis. In this section, we present the main results, focusing on the most relevant findings.

### Group comparisons

Three independent experts—a child psychiatrist and two child psychologists, each with over five years of experience—blindly agreed on the classification of 56 out of 60 participants into different ICD-11 groups. For the remaining four participants, the experts found it difficult to reach a consensus individually. After an unblinded discussion, these participants were assigned to the residual code 6A02.Z. Of these, three had normal IQs but lacked functional language, while the fourth participant had such severe behavioral difficulties that their assessment was deemed invalid. Only two participants ended in 6A02.1 group (IDD with fluent language) and were therefore excluded from the analyses using ICD-11 categories due to the small sample size. The final sample size was 54 when using ICD-11 groups, 56 otherwise. **Table 2** shows the descriptive statistics and comparisons among different ICD-11 diagnoses. The **Supplementary Data Sheet S1** provide the full descriptive analysis.

**Table 2:** Descriptive statistics and comparisons among different ICD-11 diagnoses.

Variable	6A02.0 (N = 13)				6A02.2 (N = 8)				6A02.3 (N = 8)				6A02.5 (N = 25)				Differences among	Total (N = 56)			
	Mean	SD	Mdn	Range	Mean	SD	Mdn	Range	Mean	SD	Mdn	Range	Mean	SD	Mdn	Range	Groups	Mean	SD	Mdn	Range
*Age (months)*	61.5	22.1	64	35 - 120	73.6	22.6	70	50 - 119	76.3	23.7	82	40 - 105	70.0	22.0	72	36 - 104	–	68.6	22.3	65	35 - 120
*IQ*	95.6	13.8	101	72 - 118	75.9	4.67	74.5	71 - 84	55.3	10.8	57.5	34 - 65	43.2	16.0	40	16 - 69	6A02.5 << (6A02.0; 6A02.2); 6A02.3 << 6A02.0; 6A02.3 < 6A02.2	62.4	25.1	62.5	16 - 118
*ADOS-2-CS*	5.69	0.95	5	5-8	7.25	1.58	7.5	5-9	7.50	1.07	7	6-9	7.88	1.13	8	5-10	6A02.0<< (6A02.5; 6A02.3); 6A02.0 < 6A02.2	7.18	1.42	7	5-10
*VABS-2-C*	87.7	16.6	94	45 - 106	63.8	15.5	61.5	42 - 84	55.9	20.2	49.5	38 - 87	43.6	19.0	42	20 - 80	6A02.5 << 6A02.0; 6A02.5 < 6A02.2; 6A02.3 << 6A02.0	59.5	25.1	56	20 - 106
*VABS-2-DL*	91.0	16.4	89	60 - 118	63.0	13.3	61	43 - 84	66.6	20.9	67.5	41 - 97	60.0	18.5	62	29 - 99	6A02.5 << 6A02.0; 6A02.2 << 6A02.0; 6A02.3 < 6A02.0	68.7	21.6	69.5	29 - 118
*VABS-2-S*	87.9	18.8	86	69 - 125	69.0	16.8	70	43 - 99	61.6	12.0	62	39 - 81	57.8	15.2	60	38 - 90	(6A02.5; 6A02.3) << 6A02.0; 6A02.2 < 6A02.0	67.4	19.3	65.5	38 - 125
*VABS-2-Tot*	86.2	18.8	83	53-120	56.6	16.2	59	30 - 81	50.9	11.1	52.5	36 - 66	47.6	19.2	48	20 - 81	(6A02.5; 6A02.3) << 6A02.0; 6A02.2 < 6A02.0	59.0	23.4	57.5	20 - 120
*PEP-3-C*	117.9	10.0	115	103 - 140	103.4	8.72	103	86 - 112	96.0	10.9	98	74 - 110	81.1	12.3	81	60 - 100	6A02.5 < 6A02.3 << 6A02.0; 6A02.5 << 6A02.2	94.7	17.7	98	60 - 130
*PEP-3-M*	111.5	12.8	110	92 - 140	97.9	9.95	101	83 - 107	93.0	9.61	94	77 - 110	84.4	10.4	84	60 - 110	6A02.5 << 6A02.0; 6A02.5 < 6A02.2; 6A02.3 < 6A02.0	93.3	14.0	92.5	60 - 130
*PEP-3-CB*	120.3	13.7	120	89 - 135	93.1	11.4	96	78 - 107	90.0	10.4	91	78 - 102	80.4	7.57	81	65 - 98	6A02.5 << 6A02.0; 6A02.5 < 6A02.2; (6A02.3; 6A02.2) < 6A02.0	92.5	18.2	89	60 - 135
*Age-M (years)*	38.1	4.19	39	30 - 45	43.0	4.58	44	35 - 49	40.4	2.72	41	37 - 44	39.7	8.02	41	22 - 55	–	40.0	6.30	40	22 - 55
*Age-F (years)*	42.1	4.73	42	35 - 52	45.8	4.30	47	38 - 52	41.8	6.32	40	35 - 54	44.2	7.83	44	28 - 57	–	43.6	6.58	43	28 - 57
*YoE-M (years)*	15.7	2.59	18	13 - 18	14.9	3.72	15.5	8-18	14.3	3.54	13	8-18	12.6	2.86	13	8-18	6A02.5 << 6A02.0	13.9	3.21	13	8-18
*YoE-F (years)*	14.2	3.63	13	8-18	13.0	4.63	13	8-18	12.38	1.77	13	8-18	11.8	2.61	13	8-18	–	12.5	3.14	13	8-18

Each row tests the null hypothesis that the Sample 1 and Sample 2 distributions are the same. Asymptotic significance (2-sided tests) are displayed. The significance level is.050. Differences significant after Bonferroni correction are reported as “<<“ while the one significant only before correction are reported as “<“. YoE, Years of Education.

**Figure 1:**
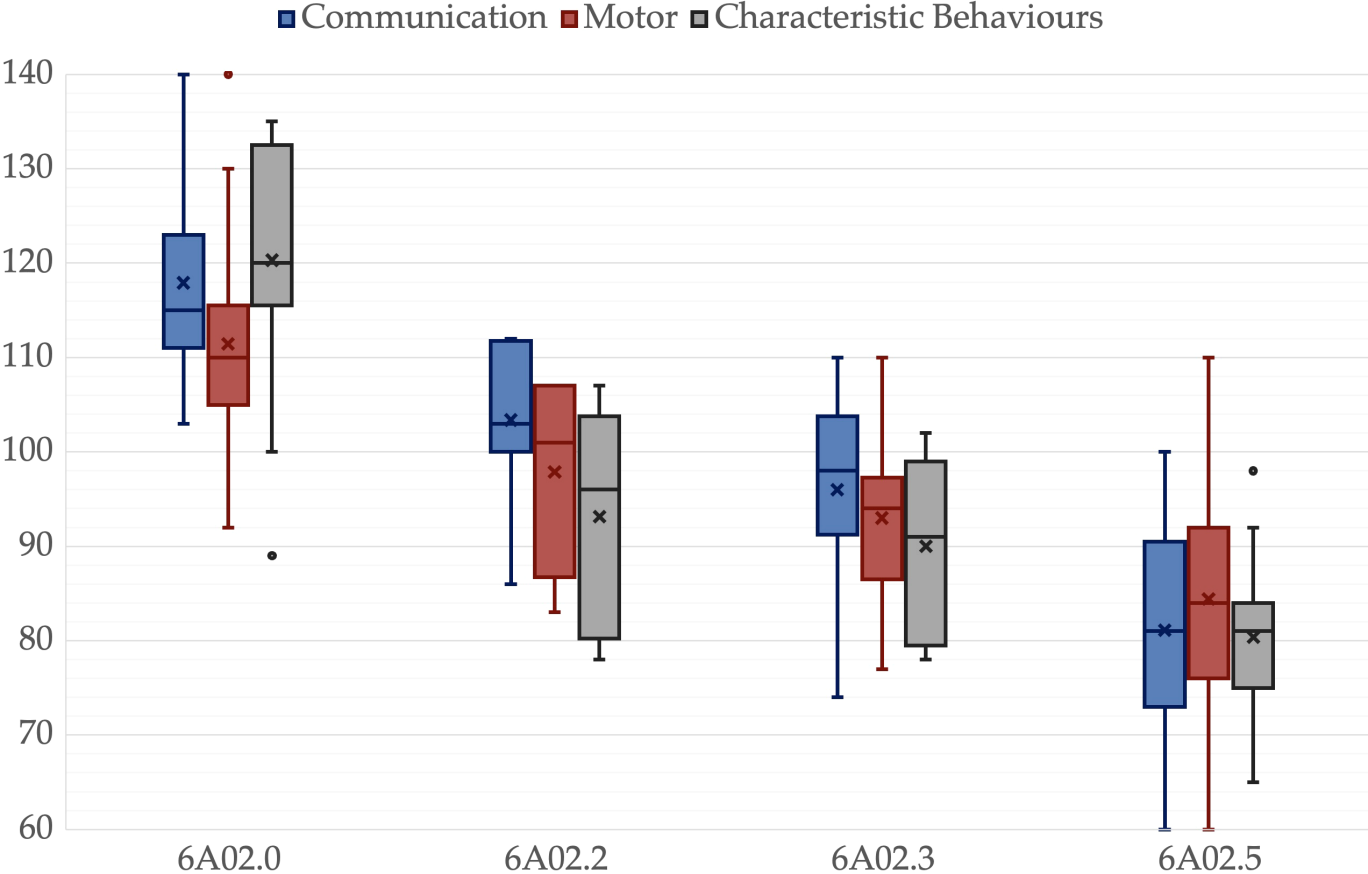
Psychoeducational Profile 3^rd^ edition scales for different ICD-11 groups. A box is drawn from the first quartile to the third quartile, while the cross is the mean value, the median is represented as a line, mean as a cross. The whiskers extend from each quartile to the minimum or maximum, outliners are presented as single points.

**Figure 2:**
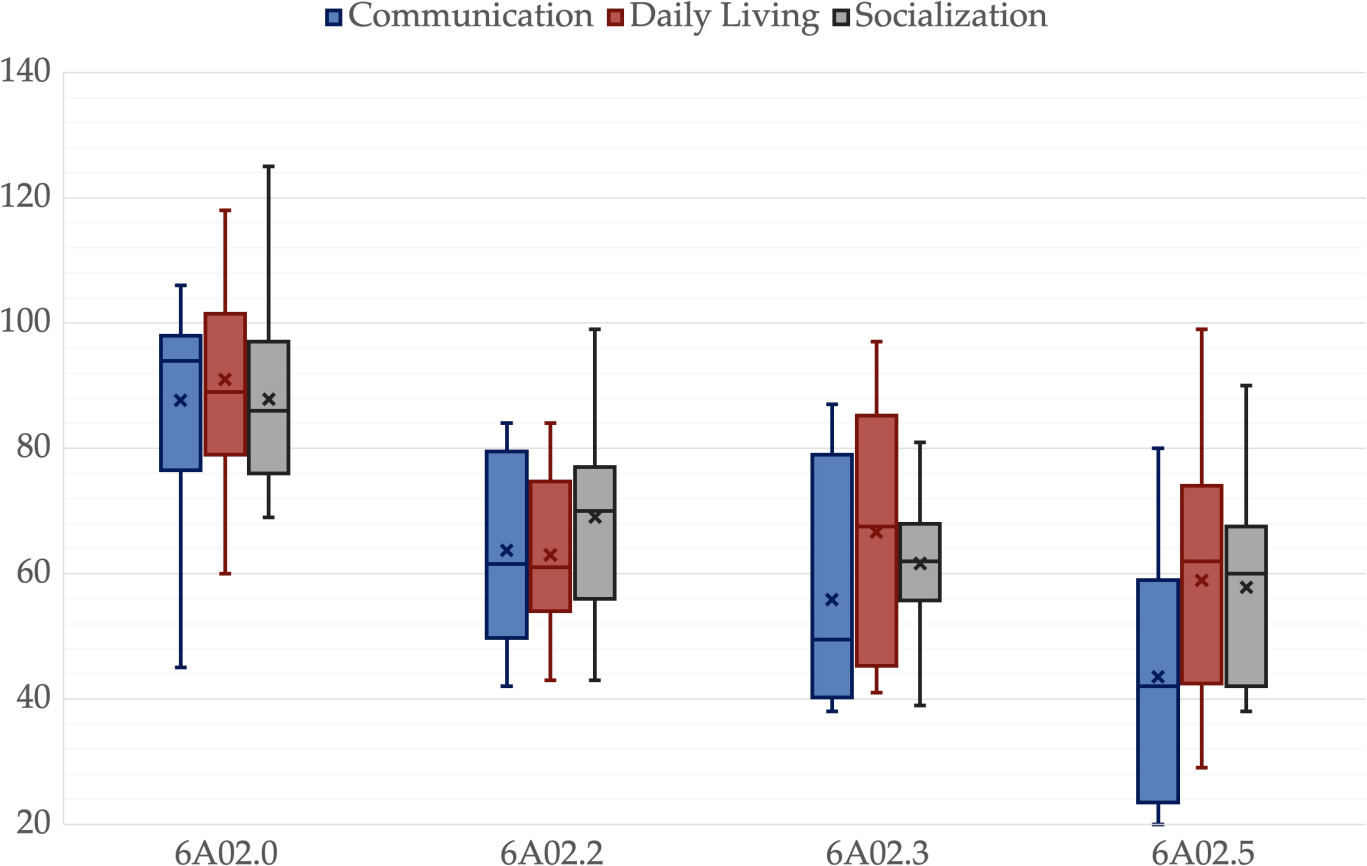
Vineland Adaptative Behavioral Scales 2^nd^ edition scales for different ICD-11 groups. A box is drawn from the first quartile to the third quartile, while the cross is the mean value, the median is represented as a line, mean as a cross. The whiskers extend from each quartile to the minimum or maximum, outliners are presented as single points.

**Figure 3:**
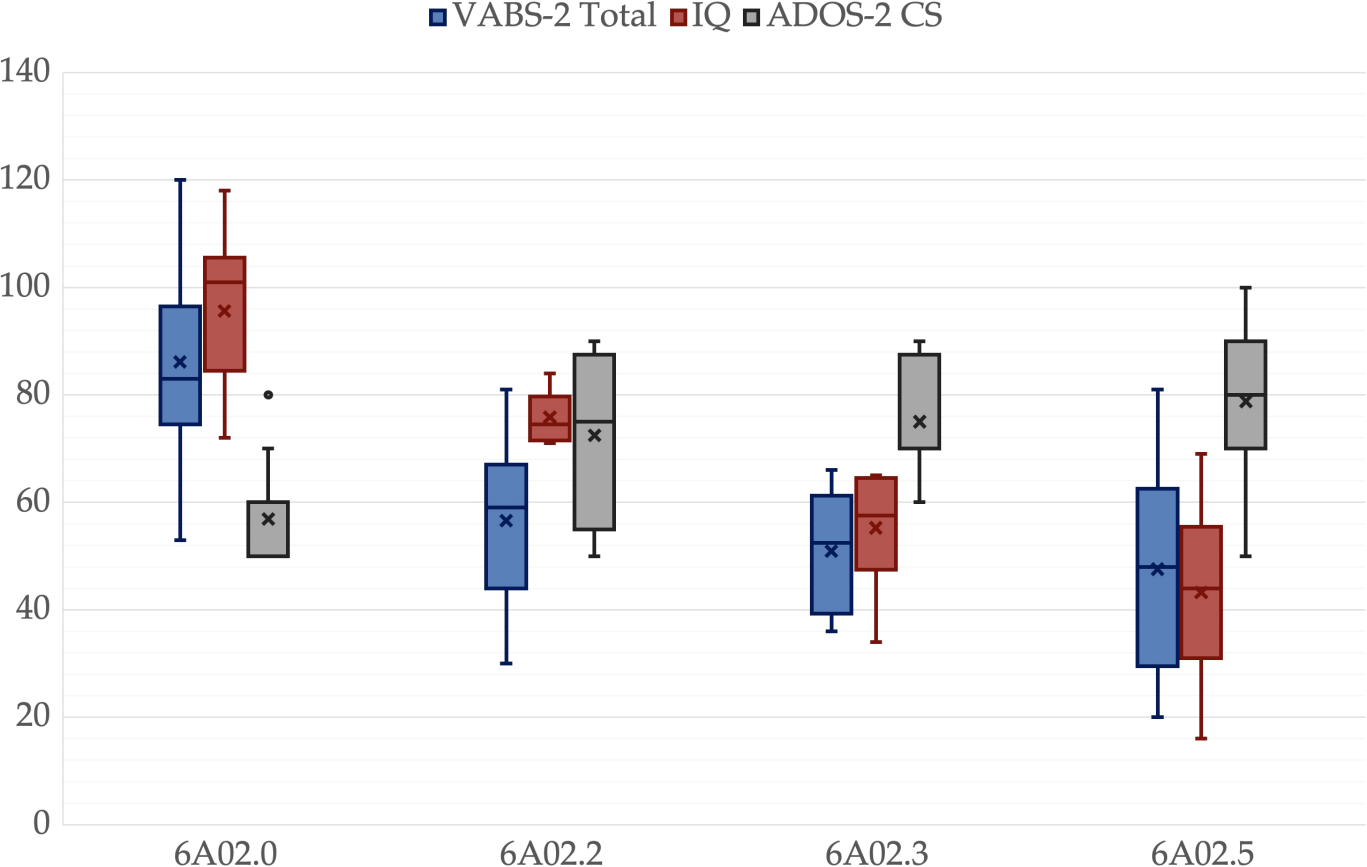
Global scales comparisons for different ICD-11 groups. A box is drawn from the first quartile to the third quartile, while the cross is the mean value, the median is represented as a line, mean as a cross. The whiskers extend from each quartile to the minimum or maximum, outliners are presented as single points. Ados-2 CS is multiplied by 10 for representation purposes.

There was no significant difference among groups in age, *p* = .325, father’s age, *p* = .214, mother’s age, *p* = .203, or father’s educational level, *p* = .214. However, there was a significant difference in the mother’s educational level, *p* = .047. All other variables showed significant differences among groups, *p* <.001. Pairwise comparisons revealed significant differences between 6A02.5 and 6A02.0 in all variables. IQ, ADOS-2-CS, VABS-2-C, VABS-2-S, VABS-2-Tot and PEP-3-C were lower in 6A02.3 than in 6A02.0, *p* = .003, *p* = .049, *p* = .025, *p* = .011, *p* = .008 and *p* = .025 respectively. IQ and PEP-3-C were greater in 6A02.2 than in 6A02.5, *p* = .001 and *p <*.001, respectively. VABS-2-DL was lower in 6A02.2 than in 6A02.0, *p* = .024. Mothers in group 6A02.0 have a higher educational level than mothers in group 6A02.5, *p* = .034. All *p*-values are adjusted for multiple comparisons. Group descriptive statistics and comparisons are reported in **Table 2**, **Figures 1**–**3** show the box plot for the clinical variables. The **Supplementary Data Sheet S2** provide the full non-parametric analysis of among groups differences.

### Correlation analysis

Correlation analysis among variables was performed first in full and then with partial correlation controlling for Age; Age and IQ; Age, IQ, and ADOS-2-CS. Age is negatively correlated with all clinical variables, *p* <.001, ranging from *r (56*) *=* -.35 for PEP-3-M and IQ to *r*(56) *=* -.55 for VABS-2-Tot, it is also positively correlated with ADOS-2-CS, *r*(56) = .39. IQ is negatively correlated with ADOS-2-CS, *r*(56) = -.64 and positively correlated with all other clinical variables, ranging from *r*(56) = .66 for VABS-2-DL to *r*(56) = .84 for PEP-3-C and PEP-3-CB, all *p* <.001. ADOS-2-CS is negatively correlated with all clinical variables, ranging from *r*(56) = -.56 for VABS-2-DL to *r*(56) = -.79 for PEP-3-C and *r*(56) = -.78 for PEP-3-CB, all *p* <.001. There is a high correlation among clinical variables, ranging from *r*(56) = .57 between VABS-2-S and PEP-3-M to *r*(56) = .88 between PEP-3-C and PEP-3-M, all *p* <.001.

All values for correlations and partial correlations controlling for Age, IQ, and ADOS-2-CS are reported in **Table 3**. Non-parametric correlations, *p*-values, and other partial correlations are reported in of **Supplementary Data Sheet S3**.

**Table 3:** Correlation and partial correlation among variables.

Variable	Age	IQ	ADOS-2 CS	VABS-2-C	VABS-2-DL	VABS-2-S	VABS-2-Tot	PEP-3-C	PEP-3-M	PEP-3-CB	YoE Parents	Age Parents	PAB
*ge*	**-**	**-.35**	**.39**	**-.44**	**-.43**	**-.35**	**-.55**	**-.37**	**-.35**	**-.38**	-.07	**.43**	.12
*IQ*	**-**	**-**	**-.64**	**.77**	**.66**	**.67**	**.71**	**.84**	**.75**	**.84**	**.34**	-.19	-.09
*ADOS-2 CS*	**-**	**-**	**-**	**-.64**	**-.56**	**-.54**	**-.64**	**-.79**	**-.69**	**-.78**	**-.28**	.23	.11
*VABS-2-C*	**-**	**-**	**-**	**-**	**.78**	**.79**	**.82**	**.81**	**.70**	**.77**	**.37**	**-.32**	-.19
*VABS-2-DL*	**-**	**-**	**-**	**.51**	**-**	**.83**	**.89**	**.64**	**.58**	**.68**	**.28**	**-.47**	**-.36**
*VABS-2-S*	**-**	**-**	**-**	**.54**	**.68**	**-**	**.85**	**.62**	**.57**	**.64**	.17	-.26	-.15
*VABS-2-Tot*	**-**	**-**	**-**	**.53**	**.76**	**.71**	**-**	**.72**	**.63**	**.75**	.23	**-.36**	-.20
*PEP-3-C*	**-**	**-**	**-**	**.38**	.08	.02	.18	**-**	**.88**	**.86**	**.39**	-.24	-.13
*PEP-3-M*	**-**	**-**	**-**	.19	.08	.06	.07	**.61**	**-**	**.77**	**.39**	-.20	-.10
*PEP-3-CB*	**-**	**-**	**-**	.24	.24	.10	**.28**	**.27**	.20	**-**	**.42**	-.19	-.09
*YoE Parents*	**-**	**-**	**-**	.18	.09	-.09	-.02	.21	.20	**.28**	**-**	.14	.18
*Age Parents*	**-**	**-**	**-**	-.17	**-.40**	-.11	-.17	-.08	-.02	.04	.23	**-**	**.95**
*PAB*	**-**	**-**	**-**	-.17	**-.40**	-.11	-.17	-.08	-.02	.03	.23	**1.0**	**-**

Correlations with p <.05 are in bold. In the upper triangle Pearson’s correlation among variables, in the lower triangle partial correlation controlling for Age, IQ and ADOS-2-CS. YoE, Parents’ Years of Education; PAB, Parental Age at Birth.

Cell colors visually represent the strength and direction of the correlations between variables. Cells shaded green indicate strong positive correlations. Cells shaded red signify strong negative correlations. Cells shaded yellow represent little to no linear relationship between the variables.

### Factor analysis

**Figure 4:**
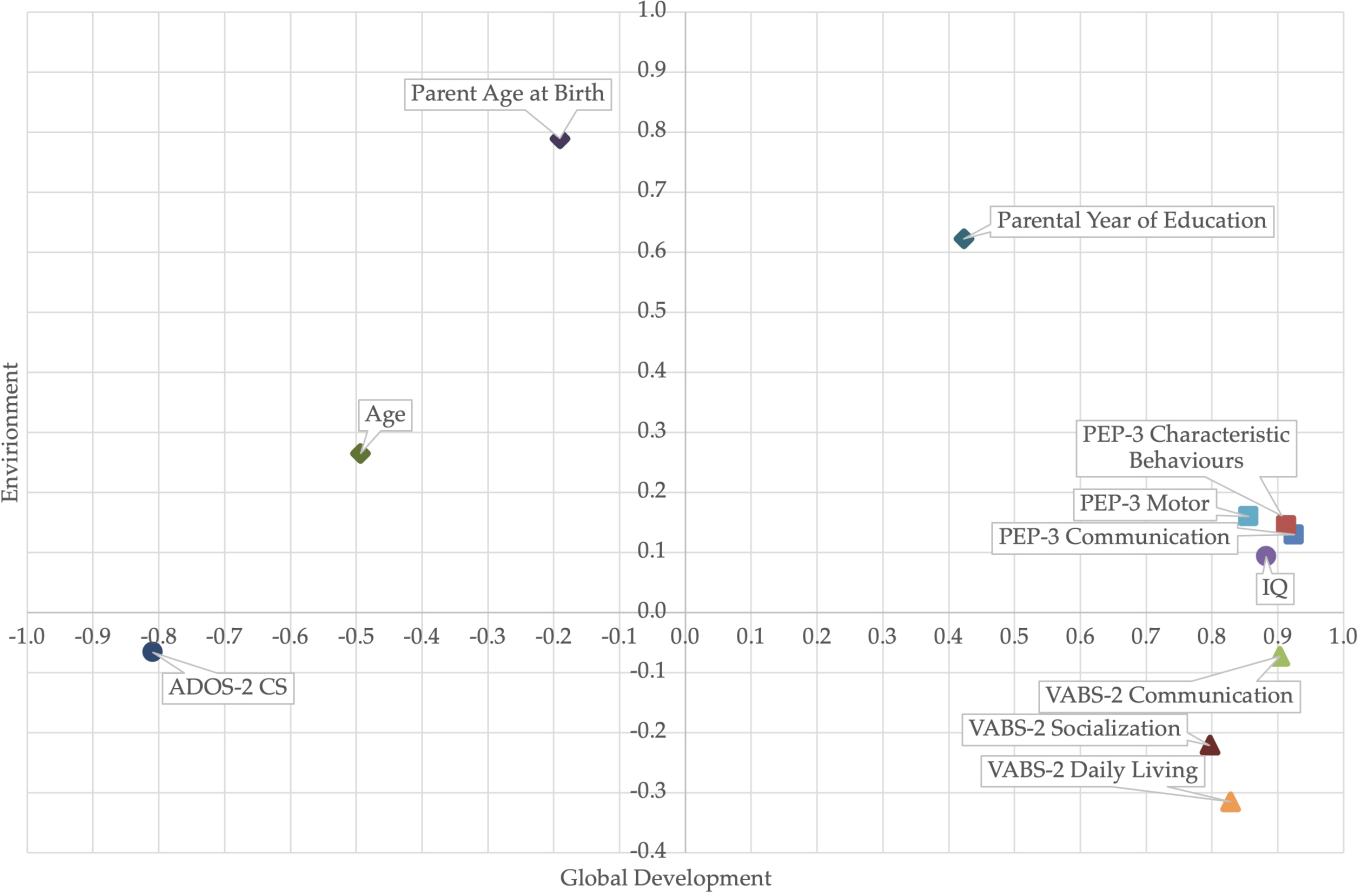
Factor analysis of developmental, adaptative, diagnostic, and environmental variables.

To disentangle the effects of different variables, we conducted an exploratory factor analysis. Even with a small sample, the Kaiser-Meyer-Olkin Measure of sampling adequacy was meritorious, *KMO* = .859, and Bartlett’s test of sphericity was significant, *p* <.001. Principal component analysis with eigenvalues greater than 1 revealed a 2-factor structure that explained 70.5% of the variance. The first rotated factor, related to Global Development, explained 58.6% of the variance, while the second factor, related to demographic (Environmental) effects, explained 11.9% of the variance. Regarding adaptive functioning and diagnostic components, they all had high loadings on the first factor [.797,.925], while VABS-2-DL and VABS-2-S loaded also on the second factor with coefficients of -.315 and -.221, respectively. For the demographic/environment component, the variable with the highest loading on the second factor was PAB,.789, along with YoE,.622. Interestingly, YoE also loaded on the first factor,.423. **Figure 4** The full analysis is reported in the **Supplementary Data Sheet S4.**

### Predictors of parent-reported adaptive functioning

The results of the multivariate tests showed that IQ had a significant effect on the dependent variables, *F*(3,49) = 15.7, *η_p_* ^2^ = .490, *p* <.001, as did PAB, *F*(3,49) = 6.45, *η_p_* ^2^ = .283, *p* <.001, and YoE, *F*(3,49) = 3.84, *η_p_* ^2^ = .190, *p* = .015. Age was marginally significant, *F*(3,49) = 2.52, *η_p_* ^2^ = .134, *p* = .069. The between-subjects effects were significant for all dependent variables in the corrected model: VABS-2-C, VABS-2-DL, VABS-2-S. PAB effect on VABS-2-DL was strong and negative, IQ had a strong positive effect on all dependent variables. When ADOS-2-CS was added in covariance, there was no effect of ADOS-2-CS and the other effects remained similar. Overall *η_p_* ^2^ increased by only.011 to.018 for different VABS-2 scales. When using ICD-11 groups as a factor, the effect of IQ was reduced, but the pattern of effects remained similar. The same held when using both ICD-11 as a factor and ADOS-2-CS in covariance (**Supplementary Data Sheet S5**).

### Predictors of directly assessed adaptive functioning

Multivariate tests, showed a significant effect of IQ, *F*(3,49) = 36.1, *η_p_* ^2^ = .688, *p* <.001, and a trend for YoE, *F*(3,49) = 1.97, *η_p_* ^2^ = .108, *p* = .130. Between-subjects effects were significant on all dependent variables for the corrected model, PEP-3-C, *F*(4,51) = 35.9, *η* ^2^ = .738, *r^2adj^* = .718, *p* <.001, PEP-3-M, *F*(4,51) = 19.1, *η* ^2^ = .600, *r^2adj^* = .568, *p* <.001, PEP-3-CB, *F*(4,51) = 35.2, *η* ^2^ = .734, *r^2adj^* = .713, *p* <.001. IQ had a strong positive effect on all PEP-3 scales; YoE had positive effect on PEP-3-CB, *F*(1,51) = 4.84, *η_p_* ^2^ = .087, *B* = 1.14, *p* = .032. When ADOS-2-CS was added in covariance, there was a high increase in model accuracy. Multivariate tests showed a significant effect of, ADOS-2-CS. Between-subjects effects was significant on all dependent variables for the corrected model and IQ had a strong positive effect on all PEP-3 scales, while ADOS-2-CS had a strong negative effect on all of them.

Using ICD-11 groups as a factor the effect of IQ was reduced but remained significant, while there was a significant effect of the ICD-11 group. Multivariate tests, showed an effect of IQ, *F*(3,44) = 13.0, *η_p_* ^2^ = .228, *p* = .009, and ICD-11, *F*(9,138) = 2.89, *η_p_* ^2^ = .159, *p* = .004. Between-subjects effects was significant on all dependent variables for the corrected model. ICD-11 classification had a an effect on PEP-3-C, *F*(3,46) = 2.89, *η_p_* ^2^ = .159, *p* = .045, and PEP-3-CB, *F*(3,46) = 7.38, *η_p_* ^2^ = .325, *p* <.001, there was no effect on PEP-3-M. *Post-hoc* pairwise comparisons showed a significant difference between 6A02.0 and all other groups on PEP-3-CB, *MD_0-2_* = 19.3, *p* <.001, *MD_0-3_* = 15.7, *p* = .034, *MD_0-5_* = 21.7, *p* = .004.

When ADOS-2-CS was added in covariance, there was a further increase in model accuracy. Multivariate tests, showed an effect of IQ, *F*(3,43) = 3.83, *η_p_* ^2^ = .211, *p* = .016, ICD-11, *F*(9,135) = 2.86, *η_p_* ^2^ = .161, *p* = .004 and a strong effect of ADOS-2-CS, *F*(3,43) = 11.38, *η_p_* ^2^ = .443, *p* <.001. Between-subjects effects was significant on all dependent variables for the corrected model. ADOS-2-CS had a strong negative effect on all PEP-3 variables while IQ had a positive effect on all of them. ICD-11 classification had a an effect on PEP-3-C, *F*(3,45) = 3.42, *η_p_* ^2^ = .186, *p* = .025, and PEP-3-CB, *F*(3,45) = 5.91, *η_p_* ^2^ = .283, *p* = .002, there was no effect on PEP-3-M.

A full analysis is reported as **Supplementary Data Sheet S6**.

### Effect of intelligence quotient on autistic behaviors

The effect of intelligence quotient on autistic behaviors was assessed using ANOVA, with ADOS-2-CS as a dependent variable. ANOVA was performed using first IQ only and then Age, PAB, and YoE as a further covariate, finally ICD-11 groups were added as factors. The test using PEP-3-CB as a dependent variable is reported in the previous chapter and therefore is not repeated here.

The corrected model using ADOS-2-CS as a dependent variable showed a significant effect of IQ, *F*(1,54) = 38.3, *η_p_* ^2^ = .415, *r^2adj^* = .404, *p* <.001. When Age, PAB, and YoE were added in covariance, the IQ effect was still significant, F(1,51) = 21.2, *η_p_* ^2^ = .294, *p* <.001, *B* = -.031, and there was a trend effect of Age, F(1,51) = 2.82, *η_p_* ^2^ = .052, *p* = .099, *B* = .012. Using ICD-11 groups as factors, IQ showed a similar effect, but it was no longer significant, 6A02.0 had a lower ADOS-2-CS score, compared to all other groups, but the difference was not significant.

When PEP-3-C was added in covariation with IQ, Age, PAB and YoE; the effect of IQ was no longer significant, F(1,50) =.348, *η_p_* ^2^ = .007, *p* = .558, while there was a large effect of PEP-3-C, F(1,50) = 25.7, *η_p_* ^2^ = .340, *p* <.001, *B* = -.064. A full analysis is reported as **Supplementary Data Sheet S7**.

### Effects of intellectual development disorder and borderline intellectual functioning

**Figure 5:**
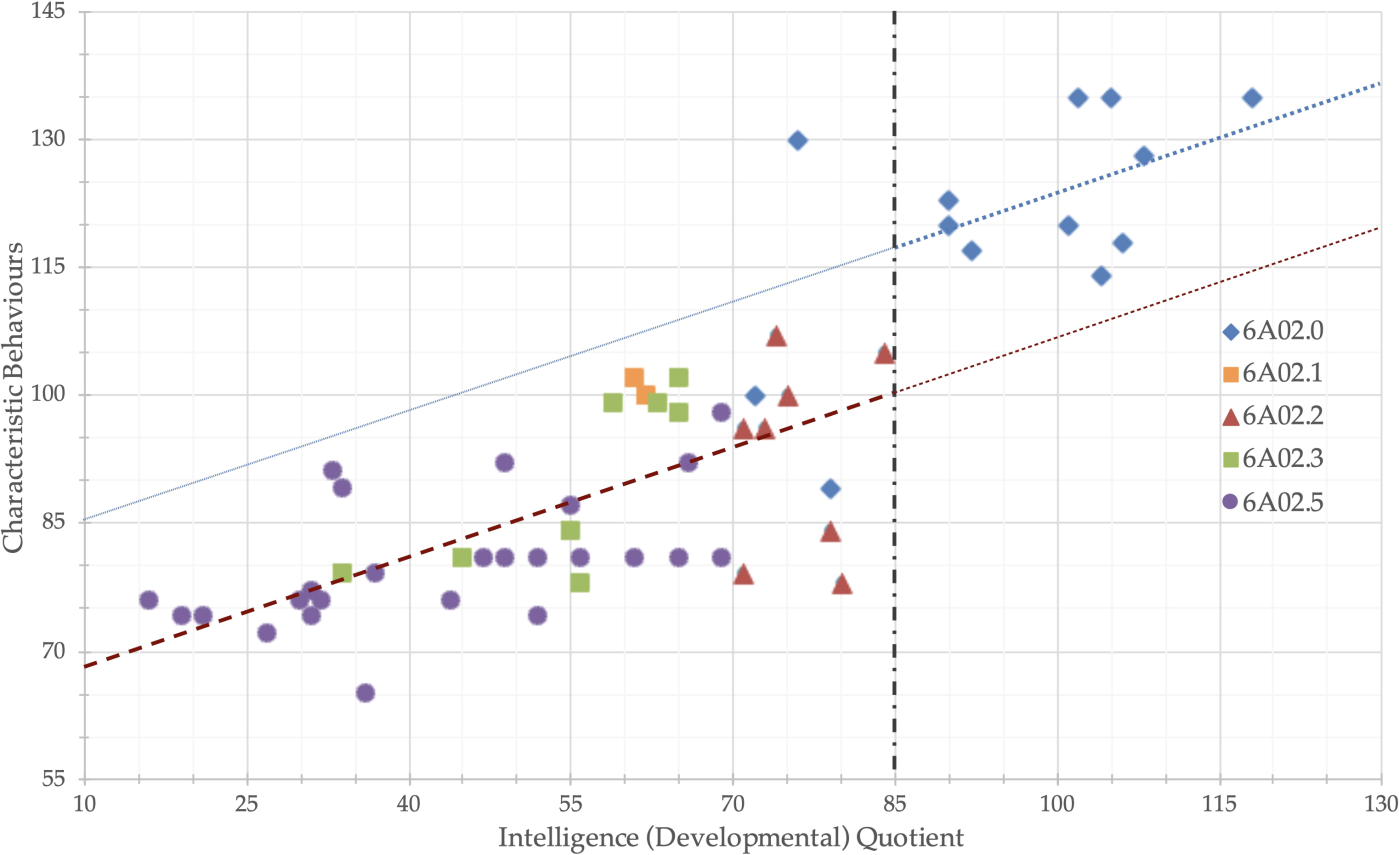
Intelligence (Developmental) Quotient and effect of language functioning on Psychoeducational Profile 3^rd^ edition - Characteristic Behaviors scale The dotted blue line is the regression line of IQ on PEP-3-CB. The dashed red line was generated by subtracting the difference on PEP-3-CB between the “Average IQ” and “Borderline IQ or less” groups. All data points with IQ > 85 are over the red line, and all data points with IQ ≤ 85, except one outliner, are below the blue line.

Instead of using the two levels presented in ICD-11 (Without IDD, IDD), we started with a finer grain analysis using Severe to Moderate ID, IQ ≤ 52, 52 < Mild ≤ 70, 71 < Borderline Intellectual Functioning (BIF) ≤ 85, and Normal Intellectual Functioning (NIF) > 85. Multivariate tests using intellectual functioning groups (IFG) as a factor, PEP-3 scales as dependent variables, and IQ, Age, YoE, and PAB as a covariate, showed a marginally significant effect of IQ, *p* = .069 and IFG, *p* = .109, but the Box’s Test of Equality of Covariance Matrices was significant, *p* = .010. A further examination showed a significant between-subject effect of IFG on PEP-3-CB, *p* = .032, but the difference between groups was not significant. Nevertheless, BIF, mild and moderate-severe (MS) IID, led to similar constants of *B_BIF_* = -14.7, *B_mild_* = -17.7, and *B_MS_* = -17.6. Therefore, we re-run the analysis with a separation into only two groups: NIF and BIF or less (BIFL). With only two levels, Box’s Test of Equality of Covariance Matrices was no longer significant, and multivariate tests showed an effect of IQ, *F*(3,48) = 17.1 *η_p_* ^2^ = .517, *p* <.001, and IFG, *F*(3,48) = 4.69, *η_p_* ^2^ = .227, *p* = .006. Between-subjects effects was significant on all dependent variables for the corrected model. IQ had a strong positive effect on all PEP-3 scales. IFG had a an effect on PEP-3-CB, *F*(1,50) = 9.42, *η_p_* ^2^ = .159, *MD*= 15.1, *p* = .003. **Figure 5** shows the different effects of IQ and IFG (two levels) on PEP-3-CB, no other factors were considered for the plot to preserve original data points, without Age, YoE, and PAB, the model parameters were *B_IQ_* = .426, *p* <.001 and *MD_IFG_* = 17.3, *p* <.001. No significant effect of IFG was found using VABS-2 scales as dependent variables when IQ was used as a covariate. A full analysis is reported as **Supplementary Data Sheet S8.**

### Effects of functional language

**Figure 6:**
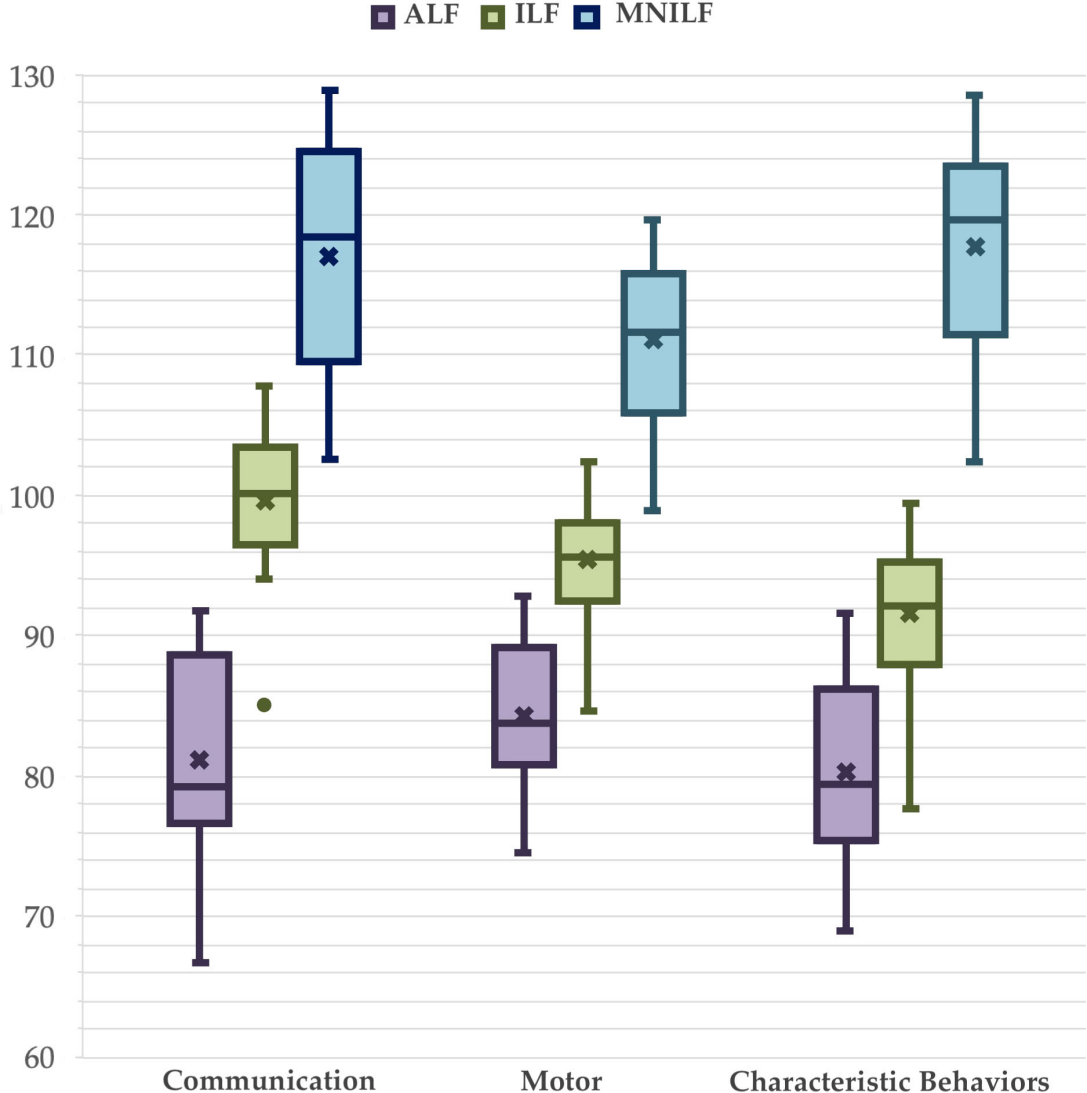
Effect of language functioning on directly assessed adaptive functioning. A box is drawn from the first quartile to the third quartile, while the cross is the mean value, the median is represented as a line, mean as a cross. The whiskers extend from each quartile to the minimum or maximum, outliners are presented as single points. Covariates appearing in the model are evaluated at the following values: Age = 68.6, IQ = 62.4, YoE = 13.4, PAB = 36.0.

For functional language (FL) three levels are present: Absence of Functional Language (AFL), Impaired Functional Language (IFL), Minimal or No Impairment in Functional Language (MNIFL), multivariate tests, using PEP-3 scales as dependent variables, showed an effect of IQ, *F*(3,47) = 7.37 *η_p_* ^2^ = .320, *p* <.001, and FL, *F*(6,96) = 4.62, *η_p_* ^2^ = .224, *p* <.001. Between-subjects effects was significant on all dependent variables for the corrected model. IQ had a strong positive effect on all PEP-3 scales. LF had a an effect on PEP-3-C, *F*(2,49) = 6.66, *η_p_* ^2^ = .214, *p* = .003, a marginal effect on PEP-3-M, *F*(2,49) = 2.66, *η_p_* ^2^ = .098, *p* = .080 and strong effect on PEP-3-CB, *F*(2,49) = 9.36, *η_p_* ^2^ = .276, *p* <.001. *Post-hoc* pairwise comparisons showed a significant difference between AFL and the two other groups on PEP-3-C, *MD_AFL-IFL_* = -11.0, *p* = .009 and *MD_AFL-MNIFL_* = -17.1, *p* = .004, and a significant difference between MINLF and the two other groups on PEP-3-CB, *MD_MNIFL-IFL_* = 15.3, *p* <.001 and *MD_MNIFL-AFL_* = 18.7, *p* = .001 (**Figure 6**). Using VABS-2 scales as dependent variables when IQ or IQ and ADOS-2-CS were in covariate, while without IQ and ADOS-2-CS, the multivariate effect of FL was significant, *F*(6,98) = 4.62, *η_p_* ^2^ = .220, *p* <.001 as all the Between-subjects effects.

In a second ANOVA analysis, we studied the effect of FL on IQ. Between-subjects effects were significant for the corrected model, *F*(6,49) = 18.7, *η* ^2^ = .696, *r^2adj^* = .658, *p* <.001. The effect of FL was very large, *F*(2,49) = 19.1, *η_p_* ^2^ = .439, *p* <.001 and there was a significant difference (*p* <.001) among all three groups, after accounting for demographic variables and ADOS-2-CS, *M_AFL_* = 46.2, *M_IFL_* = 67.5 and *M_MNIFL_* = 84.1.

Finally, we also introduced PEP-3-C in covariance. There was no ADOS-2-CS effect on IQ, but there was a large effect of PEP-3-C, *F*(1,48) = 11.2, *B* = .804, *η_p_* ^2^ = .189, *p* = .002, and the effect of FL was still present, *F*(2,48) = 3.64, *η_p_* ^2^ = .132, *p* = .034.

A *post-hoc* analysis, the reason for which will be explained in the discussion, was performed to study internal differences of the AFL group with a subdivision based on Age Group (AG) between younger (AFL-Y; Age < 72 months) and older children (AFL-O), this *post-hoc* analysis also included participants classified as 6A02.Z (a second analysis without them led to similar results). There was a strong association among all adaptive and diagnostic variables (except VABS-2-S which was trending but non-significant) and group membership, with a higher score (lower for ADOS-2-CS) in AFL-Y. AFL-Y had an average age of 41 months while AFL-O was 82 months, there was no difference in YoE or PAB but IQ was 58.8 in AFL-Y and 40.8 in AFL-O, *p* = .009; PEP-3-C was 92.8 in AFL-Y and 77.4 in AFL-O, *p* = .001; PEP-3-CB was 91.3 in AFL-Y and 78.5 in AFL-O, *p* = .004; VABS-2-C was 54.7 in AFL-Y and 39.4 in AFL-O, *p* = .036; VABS-2-Tot was 65.3 in AFL-Y and 40.2 in AFL-O, *p* = .001. Furthermore, also the three subscales of PEP-3-C (preverbal/verbal IQ, expressive and receptive language) were significantly different, *p* <.019. *A* full analysis is reported as **Supplementary Data Sheet S9**. 

### Predictive value of parental assessment of adaptive functioning on direct assessment

PEP-3 scales were used as dependent variables, while VABS-2 scales were used as covariates.

Multivariate tests showed a significant effect of VABS-2-C, *F*(3,50) = 10.9, *η_p_* ^2^ = .396, *p* <.001. Between-subjects effects were significant on all dependent variables for the corrected model. VABS-2-C had a strong positive effect on all PEP-3 scales.

Adding IQ, Age, YoE, and PAB in covariate, VABS-2-C was still significant, *F*(3,46) = 3.39, *η_p_* ^2^ = .181, *p* = .026, and also IQ had a significant effect, *F*(3,46) = 11.8, *η_p_* ^2^ = .435, *p* <.001,. Between-subjects effects were significant on all dependent variables for the corrected model. VABS-2-C had a strong positive effect on PEP-3-C, *F*(1,48) = 9.71, *η_p_* ^2^ = .168, *B* = .323, *p* = .003, while IQ had a strong effect on all PEP-3 scales.

Adding LF as a factor, VABS-2-C became barely significant, *F*(3,44) = 2.49, *η_p_* ^2^ = .145, *p* = .072, IQ had still a significant effect, *F*(3,44) = 4.00, *η_p_* ^2^ = .214, *p* = .013, and there was a significant effect of LF, *F*(6,90) = 3.70, *η_p_* ^2^ = .198 *p* = .002. Between-subjects effects was significant on all dependent variables for the corrected model. VABS-2-C had a positive effect on PEP-3-C, *F*(1,46) = 7.00, *η_p_* ^2^ = .132, *B* = .260, *p* = .011, while IQ had an effect on all PEP-3 variables. Furthermore, LF had a significant effect on PEP-3-C, *F*(2,46) = 4.87, *η_p_* ^2^ = .175, *p* = .012 and PEP-3-CB, *F*(2,46) = 7.74, *η_p_* ^2^ = .252, *p* = .001. *Post-hoc* pairwise comparisons showed a significant difference between AFL and the two other groups on PEP-3-C, *MD_AFL-IFL_* = -9.49, *p* = .026 and *MD_AFL-MNIFL_* = -14.3, *p* = .017, and a significant difference between MINLF and the two other groups on PEP-3-CB, *MD_MNIFL-IFL_* = 14.4, *p* = .002 and *MD_MNIFL-AFL_* = 17.4, *p* = .003. Adding ADOS-2-CS as a covariate, the multivariate effect of VABS-2-C was no longer significant, *p* = .090, but the univariate effect on PEP-3-C was still significant, *p* = .016. The pattern of all other effects was similar. Between-subjects effects was significant on all dependent variables for the corrected model; ADOS-2-CS effect was significant for all PEP-3 scales.

A full analysis is reported as **Supplementary Data Sheet S10.**

To assess the predictive value of the total VABS-2 scale (comprising also motor skills for children younger than six), PEP-3 scales were used as dependent variables, while VABS-2-Tot was used as a covariate.

Multivariate tests showed a significant effect of VABS-2-Tot, *F*(3,52) = 24.5, *η_p_* ^2^ = .585, *p* <.001. Between-subjects effects were significant on all dependent variables for the model.

Adding IQ, Age, YoE, and PAB in covariate, VABS-2-Tot was still significant, *F*(3,48) = 3.02, *η_p_* ^2^ = .159, *p* = .039, and IQ had a strong significant effect, *F*(3,48) = 15.4, *η_p_* ^2^ = .491, *p* <.001, Between-subjects effects was significant on all dependent variables for the corrected model. VABS-2-Tot had a positive effect on PEP-3-C, *F*(1,50) = 4.37, *η_p_* ^2^ = .080, *p* = .042, and PEP-3-CB, *F*(1,50) = 8.35, *η_p_* ^2^ = .143, *p* = .006, while IQ had a strong positive effect on all variables. Multivariate test for YoE was not significant but “trending”, with *F*(3,48) = 2.04, *η_p_* ^2^ = .113, *p* = .121, the effect was positive on all outcome variables.

Adding LF as a factor, and the ADOS-2-CS as a covariate, the VABS-2-Tot effect was no longer significant, but the direction and magnitude of the VABS-2-Tot, IQ, and YoE effect were similar. The effects of LF and ADOS-2-CS were similar to the ones for the previously reported VABS-2 subscales model.

A full analysis is reported as **Supplementary Data Sheet S11.**

## Discussion

We conducted a comprehensive study involving a diverse group of autistic children. We collected various types of data, including demographic variables such as the child’s age (Age), parental age at childbirth (PAB), and parents’ years of education (YoE). Additionally, we utilized diagnostic measures, specifically IQ and ADOS-2 comparative scores (ADOS-2-CS), along with the language functioning category (LF), to evaluate ICD-11 classification and intellectual development disorder (IDD). To assess adaptive functioning, we employed two approaches: (1) parent reports, we used the Vineland-II Adaptive Scales (VABS-2) based on information provided by parents; (2) direct assessment, a professional evaluated adaptive functioning using the Psychoeducational Profile 3rd edition (PEP-3). Both VABS-2 and PEP-3 consisted of three major subscales: Communication (C), assessed in both VABS-2 and PEP-3; Social (S) and Daily Living (DL), evaluated using VABS-2; Motor (M) and Characteristic/Maladaptive Behaviors (CB), assessed through PEP-3. Additionally, VABS-2 included a motor scale, but it applied only to children under the age of six and contributed to the overall VABS-2 total score.

In our study, we conducted a Principal Component Analysis (PCA) on a dataset with eigenvalues greater than 1. The analysis revealed a two-factor structure that collectively explained 70.5% of the variance. The first factor, which accounted for 58.6% of the variance, is closely related to global developmental aspects. Clinical components such as IQ and ADOS-2 CS (negative), and both adaptive functioning scales exhibited high loadings on this factor, with coefficients ranging from 0.797 to 0.925. The second factor, explaining 11.9% of the variance, is associated with demographic effects. Notably, PAB had the highest loading on this factor (.789), followed by YoE (.622). YoE also loaded on the first factor (.423) showing a contribution of parental education on global development.

### Effects of demographic

We found effects of Age, YoE, and PAB on different outcomes. We observed a weak to moderate negative correlation between age and all diagnostic variables (positive for ADOS-2-CS) as well as adaptive behavior variables.

Although there were no significant age differences among the ICD-11 groups in our sample, a deeper analysis of IQ levels revealed an interesting pattern. Children with an IQ greater than 85 were significantly younger than those with borderline, moderate, and severe IDD (*p* = .033). Consequently, we cannot dismiss the possibility that this effect may be influenced by sample bias, at least for certain variables. Interestingly, the negative effect of age on the VABS-2 total score persisted even after accounting for covariance with other demographic variables, IQ, ADOS-2-CS, and PEP-3-CB. However, given that the VABS-2 total score is computed differently for children older and younger than 6 years of age, we re-ran the analysis separately for these two age groups, and the effect completely disappeared (even without other variables as covariates). In future research, it would be important to investigate whether the age cutoff effect observed in the VABS-2 total score is solely an artifact of computational methods and sampling or if it reflects maturational or social factors (such as starting school). For instance, Hill and colleagues (37) found that intellectual functioning and ASD symptom severity moderated the relationship between age and adaptive functioning. ASD symptom severity was associated with better adaptive functioning in younger children with lower intellectual functioning and older children with higher intellectual functioning. Therefore, extending the sample to older ages and examining the interaction between autistic traits and IQ could provide valuable insights into the complex interplay among age, intelligence, autistic traits, and adaptive functioning.

The education level of parents exhibited a weak to moderate correlation (negative for ADOS-2-CS) with all diagnostic and adaptive functioning variables, except for the VABS-2 social and total scale. When controlling for other demographic and diagnostic factors, these correlations were halved and the only remaining significant was with PEP-3-CB (*r* = .28). Further analyses revealed that IQ had an impact on all variables. The relationship between parental years of education (YoE) and IQ found in this study, aligns with existing literature (38) and is probably mediated by both IQ heritability and childhood education (39). After accounting for IQ and other confounding factors, in MANOVA analyses we observed a positive effect of YoE on Characteristic Behaviors and a marginally positive effect on Communication (both PEP-3 and VABS-2 scales) and Motor skills. The impact of parental education on various adaptive functioning variables, independent of IQ, underscores the importance of educating parents. Targeted parental training programs could play a crucial role in enhancing these outcomes.

The age of parents at childbirth (PAB) exhibited a moderate negative correlation with Daily Living skills. This relationship persisted even after accounting for all the other diagnostic and demographic factors. Surprisingly, for each additional year of parental age, there was approximately one point less on the VABS-2-DL scale. Our literature search did not yield relevant results, so we informally consulted eight expert clinicians. Their main suggestion was that overprotection by older parents might mediate a slower acquisition of DL skills in young children. Although we found no existing research on overprotection, adaptive functioning, and parental age, we believe it is crucial to plan studies in this area. If the finding is confirmed, investigating the specific mechanisms behind the correlation can inform interventions. If the suggested mechanism accumulates evidence, it would be important to develop targeted training programs that could help parents enhance their child’s autonomy while raising awareness about the balance between protection and fostering independence.

### Effects of intelligence and global development

In our study, intelligence was primarily assessed using the Griffith-III Developmental Quotient, which constituted 80% of the final sample. As expected, IQ demonstrated a strong correlation with all adaptive functioning scales (*r* =.66 to 84). In multivariate analysis, the effect of IQ was substantial, explaining a significant portion of the variance across all adaptive scales, particularly for PEP-3 Communication and Characteristic Behaviors. Additionally, IQ showed a robust negative correlation with ADOS-2-CS (*r* = -.64). When we included ADOS-2-CS as a covariate, the variance explained by IQ decreased by approximately 20% for the PEP-3 scale, but it remained substantial, and the effect on VABS-2 scale was minimal.

We also investigated the possibility of a categorical effect related to Intellectual Functioning Groups (IFG) in addition to the continuous effect. Our analysis did not reveal a significant categorical distinction based on the presence or absence of Intellectual Development Disorder (IDD) when accounting for the linear effect of IQ. However, when we set the cutoff at the Borderline Intellectual Functioning level (BIF, IQ = 85) instead of the IDD level (IQ = 70), a very pronounced categorical effect emerged. For the multivariate analyses using PEP-3 scales, IFG explained an impressive *η_p_* ^2^ of 0.23, while IQ accounted for *η_p_* ^2^ of 0.52. Specifically, IFG had a substantial impact on PEP-3 Characteristic Behaviors (*η_p_* ^2^ = 0.16). **Figure 5** illustrates the linear effect of IQ on Characteristic Behavior: for every 37 IQ points (equivalent to 2.5 standard deviations), there is a 15-point increase of PEP-3-CB. Interestingly, transitioning from borderline to normal intellectual functioning (a one-standard-deviation increase in IQ) results in an additional one-standard-deviation increase in Characteristic Behavior scores.

The observation of a step effect at the Borderline Intellectual Functioning (BIF) level did not catch us entirely off guard; recently Katusic and colleagues (40) in a large epidemiological study found an increase in medical costs and an increase in clinical diagnoses compared to a population sample in children with IQ < 85. Throughout the history of autism research, the spotlight has predominantly illuminated two groups: individuals with Intellectual Development Disorder (IDD) and those often labeled as “high-functioning” (HFA), encompassing those with borderline, average, or above-average IQ. However, when it comes to understanding cognition and daily functioning in individuals with autism who specifically fall within the BIF range, the literature remains frustratingly sparse. Recently, a significant step was taken in the form of “The Girona Declaration on Borderline Intellectual Functioning” (41). This declaration serves as a rallying cry, emphasizing the urgent need for targeted research and intervention for this unique population. Among the scant existing studies that delve into autism and BIF, Barnevik and colleagues (42) reported a high prevalence of comorbidities. Furthermore, half of the children experienced either a significant decline, in the range of IDD, or an increase, in the range of average intellectual functioning, since their previous assessments upon entering school.

When the DSM-5 merged Asperger Syndrome (AS) into the broader ASD category, it wasn’t solely due to the challenges in distinguishing between AS and ASD with IDD or impaired language. Instead, the crux lies in the ambiguity surrounding the differentiation between HFA and AS. The latter group was characterized by the absence of developmental or language delays during early development. Despite statistical significance found in systematic reviews (43), meta-analytical studies (44), and genetic investigations (45), the AS-HFA boundary remained blurred, and the distinction lacked clarity and consistency in clinical practice and research.

The term HFA was often used in scientific articles, research designs, and everyday communication to refer to autistic individuals without IDD (46–51). However, this term was problematic for several reasons. First, it ignored the discrepancy between cognitive and adaptive functioning that is common in autism (22, 25, 52), which may mask the diverse needs and challenges of autistic individuals across different domains of life. Second, it seemed to imply a better prognosis and quality of life for those with HFA, which is not always the case. Indeed, many studies have shown that HFA is not associated with better outcomes in terms of social, emotional, or mental health (53, 54). Third, it overlooks the heterogeneity and variability of the autism spectrum, which cannot be captured by a single binary division on a single dimension. For instance, Alvares and colleagues (22) found that the gap between IQ and Vineland Adaptive Behavior Scales (VABS) scores was larger for children without IDD than for those with IDD and that this gap did not diminish with age. In our sample, higher IQ predicted a higher adaptive score on all scales, but there was a moderate negative correlation between the ratio of VABS-2-Tot over IQ, and IQ, *r (* 56) = -.459, *p* <.001, and the same ratio and Age once controlled for IQ, YoE and PAB, *r (* 53) = -.374, p = .005, showing an increase in AF - IQ gap with age and IQ.

We do not advocate for a return to the old categorical divide between Low Functioning Autism (LFA), HFA, and AS. However, we believe that the current subdivision presented in the ICD-11 is unsatisfactory, and the absence of such a subdivision in DSM-5-TR is even more concerning. When DSM-5 was proposed, merging the Spectrum into a single category was seen as an opportunity to free researchers from stringent diagnostic chains and encourage more meaningful subtyping (55). Although subtyping can evoke negative connotations, often associated with stereotyping and marginalization, it can also help identify shared genetic variants associated with specific medical issues and facilitate tailored support. Unfortunately, clinicians and researchers began using a single diagnostic label without specification, oversimplifying, and losing the meaning of the acquired freedom.

Therefore, we propose to start with a critical understanding of the literature. When reporting older studies, many researchers amalgamate previous findings on AS, HFA, ASD, etc. on the idea that the distinction is “no longer valid”. On the contrary, we recommend meticulously selecting old but well-characterized studies that explicitly report IQ ranges and language development. These studies can serve as an initial stepping stone toward unraveling the intricate effects of IQ on adaptive function. Furthermore, when studying intellectual functioning in ASD, mild, moderate, and severe IDD should be examined separately to better understand the nature of profound autism. Similarly, when studying ASD without IDD, at least three separate groups should be distinguished: borderline, average, and gifted.

### Effects of language

As anticipated, the presence, impairment, or absence of functional language significantly influenced IQ scores. Notably, the differences were substantial, exceeding one standard deviation. The AFL group fell well within the moderate IDD range, participants in the IFL group were at the cut-off between mild IDD and BIF, and individuals in the MNIFL group were at the cut-off between BIF and average intelligence. When we considered IQ, ADOS-2-CS, and demographic variables together, there was a difference between AFL and the other groups on the PEP-3 Communication scale (AFL had lower scores than IFL and MNIFL), and a difference among MNIFL and the other groups on the Characteristic Behaviors scale (MNIFL had a higher score than IFL and AFL). Even in those cases, the differences were of a standard deviation magnitude.

Unfortunately, we couldn’t compute an interaction effect between functional language and intellectual functioning due to the dimension and composition of our sample. However, it’s worth noting that all children in our sample IQ > 85 exhibited minimal to no impairment in functional language (MNIFL).

Three out of four of the excluded children in our study were not assigned to a specific ICD-11 group because they were without IDD but with MNIFL. In addition to the five ICD-11 groups, a few studies have suggested the presence of a sixth group characterizing AFL children without IDD, 16% in Bal and colleagues (56). Nevertheless, all three children in our group had BIF with an IQ between 72 and 75 and important co-occurring conditions, reinforcing the importance of considering the presence of BIF as a useful specifier. Furthermore, two of the excluded children were very young; many children in our AFL group may develop vocal language in the future. Previous studies suggested that valuable discrimination of outcome based on language development is not consistent at an early age and will develop later. Moreover, the third one probably had a significant co-occurring speech sound disorder (SSD) with the presence of good communication and receptive language skills. A small subgroup of AFL children with that profile was described by Broome and colleagues (57). In our opinion, those children are better classified as IFL or MNIFL plus SSD, instead of AFL.

In a *post-hoc* analysis of AFL, we divided children into a younger and older group with a cut-off at age six. Younger children had higher scores on all communication skills, characteristics, behavior, VABS-2 total, and IQ, and lower ADOS-2-CS scores. On average, their skills were more comparable with older children classified as 6A02.3 than with older children in the 6A02.5 group. Is it possible that many of these children don’t have functional language “yet” and will go on developing it? We are planning to follow them longitudinally to keep track and report their development.

### Effects of autistic traits

Autistic Traits (AT) were assessed using the ADOS-2-CS and PEP-3-CB. Individuals with higher AT tend to exhibit higher ADOS-2-CS scores and lower PEP-3-CB scores. Notably, both variables demonstrated a strong correlation with IQ and all adaptive functioning scales. Specifically, the correlation coefficients ranged from -0.54 to -0.78 for ADOS-2-CS and from 0.64 to 0.86 for PEP-3-CB.

When IQ was controlled for, the effect of ADOS-2-CS decreased but there were still many significant effects. There was no additional effect of ADOS-2-CS on VABS-2 scales but a large effect size on PEP-3 scales, *η_p_* ^2^ = .44, with each increase in ADOS-2-CS, decreasing Communication and Characteristic Behaviors by 5 points and Motor skills by three and a half, the effect was preserved using LF, IFG or ICD-11 groups. The relation of PEP-3-CB with IQ and other variables was reported in the previous sections.

Recently, a large longitudinal study of very young children (58) established a relationship between IQ and the ADOS-2 Calibrated Severity Score. This study revealed that lower IQ in children aged 2 to 68 months precedes an autism diagnosis by approximately 2–4 years and is associated with greater ADOS-2-CS. In our sample, for every two standard deviations decrease in IQ, ADOS-2-CS decreases by one point, while PEP-3-CB increases by 15 points (equivalent to 1 standard deviation). The ADOS-2 Comparative Score ranges from 4 to 10 in children with ASD, and from 1 to 3 in children without an ASD diagnosis. Even a single point difference is clinically significant. Interestingly, Wolff and colleagues (59) reported a three-fold increase in false positives among children with Intellectual and Developmental Disabilities (IDD) and a 1.7-fold increase in false negatives in children with IQ > 115. Johnson and colleagues (60) found that higher verbal intelligence was most associated with less ADOS-2-CS and the association was driven by communication symptoms. In our sample, when PEP-3-C was added in covariance, the association between IQ and ADOS-2-CS disappeared, and PEP-3-C explained a third of the total variance. Given the relevance of verbal intelligence in predicting autism symptom severity, subtyping autism on the basis of verbal intelligence could lead to more personalized treatments (60). Furthermore, with the robust correlation between IQ and ADOS-2-CS, there is an urgent need to establish better diagnostic instruments (or cut-offs) to prevent misdiagnosis in individuals with IDD and to ensure appropriate diagnosis and support for those with high intelligence (61).

### Relation between reported and direct assessment of adaptive behaviors

We investigated the relationship between reported adaptive behaviors by parents during VABS-2 interviews and directly measured adaptive scales using PEP-3. Notably, the Communication and Total scales of VABS-2 were the only significant contributors (in separate analyses). Both VABS-2-C and VABS-2-Tot had a substantial impact on all PEP-3 scales, with a less pronounced effect on the Motor scale. As expected, VABS-2-C had a greater influence on PEP-3-C. Interestingly, VABS-2 Socialization and Daily Living skills did not significantly affect PEP-3 scales, as they measure different constructs.

When we introduced IQ and demographic variables into the covariance analysis, the effect of VABS-2-C remained significant only for PEP-3-C, and the explained variance was halved. Simultaneously, the effect of VABS-2-Tot was also reduced but remained substantial for PEP-3-CB. The effect of VABS-2-C on PEP-3-C persisted even after including LF and ADOS-2-CS in the covariance analysis. LF explained the same amount of variance as VABS-2-C.

### ICD-11 classification

As expected, 6A02.0 and 6A02.5, the group without IDD (WIDD) and language impairment, and the group with IDD and without functional language, were statistically different on all variables. Furthermore (1): VABS-2-DL was lower in 6A02.2 (WIDD, IFL) than in 6A02.0; (2) IQ, ADOS-2-CS, VABS-2-C, VABS-2-S, VABS-2-Tot, and PEP-3-C were lower in 6A02.3 (IDD, IFL) than in 6A02.0; (3) IQ and PEP-3-C also showed differences between 6A02.3 and 6A02.0.

However, it’s worth noting that many of the differences between 6A02.0 and both 6A02.2 and 6A02.3, as well as between them and 6A02.5, may not have been statistically significant due to the small sample size. Interestingly, we found no significant difference between 6A02.2 and 6A02.3.

The distinction among different groups on the variables we selected seems mainly governed by language functioning. While factoring in LF made a large difference, the IDD or WIDD distinction didn’t contribute significantly beyond the linear contribution of IQ. Furthermore, in our sample, 6A02.2 and 6A02.3 had similar IQs. The median difference was 17 IQ points with the same IF, whereas the difference between 6A02.0 and 6A02.2 was 27 IQ points and one level of IF, and the difference between 6A02.3 and 6A02.5 was 18 IQ points and one level of IF.

When we used IQ and demographic variables as covariates, ICD-11 classification had no statistically significant effect on VABS-2 scales. However, there was an effect on PEP-3-C and PEP-3-CB, independently of ADOS-2-CS presence in covariance. *Post-hoc* comparisons revealed: (1) a significant difference between 6A02.0 and all other groups on PEP-3-CB; (2) a difference between 6A02.3 and 6A02.5 on PEP-3-C; (3) no difference among 6A02.3, 6A02.2, and 6A02.0 on PEP-3-C (p >.87 for all combinations).

The difference between 6A02.0 and other groups on PEP-3-CB was 15 points (1 SD), while the difference between 6A02.5 and other groups on PEP-3-C was 10 points.

The pattern of differences mirrors that observed for LF, suggesting that the effects of ICD-11 classification are primarily driven by LF differences rather than the presence or absence of IDD. The absence of a categorical effect of IDD raises doubts about the usefulness of the ICD-11 classification. In our view, a classification system should provide additional information beyond what continuous variables, or the sum of different diagnoses can offer. Perhaps a finer IQ level, measured in 15-point intervals, spanning the lower and higher ends of the spectrum, could be more suitable initially until more informative and ‘natural’ cut-offs are identified.

The findings from our study, particularly regarding language functioning, resonate with the results reported by Georgiou and Spanoudis (62). They demonstrated that within the ASD population, a subgroup exists that presents with language impairments similar to those seen in children with Developmental Language Disorder (DLD). This overlap suggests that language difficulties in autism may be multifaceted—due not only to general developmental factors like low IQ or to autism-specific language difficulties but also to the co-occurrence of DLD.

Unfortunately, both the DSM-5 and ICD-11 do not allow for the diagnosis of DLD in comorbidity with ASD, which poses a challenge for both research and clinical practice. This rigid separation fails to capture the complexity of language difficulties in autism, which may arise from multiple factors, including intellectual disability, autism-specific impairments, and comorbid DLD. As Georgiou and Spanoudis (62) argue, the existence of a subgroup of ASD individuals with language impairments similar to those in DLD suggests that treating these as mutually exclusive diagnoses limits our understanding of the full range of language difficulties in ASD. This is an area where both diagnostic frameworks could benefit from revision to allow for more nuanced classifications that better reflect the complex interplay between language, cognitive functioning, and ASD.

### Limitation

One key limitation of this study is the relatively small sample size (N = 60), which becomes more significant when dividing the participants into smaller subgroups based on variables like IQ, language abilities, and diagnostic categories (e.g., ICD-11 classifications). Although our statistical analyses were robust, the reduced size in each subgroup limits the generalizability and statistical power of the findings. This can affect the ability to detect subtle effects or interactions and makes the results more susceptible to variability.

Additionally, the specialized nature of the sample, drawn from a clinical setting, may not fully represent the broader population of individuals with ASD). While this is a constraint inherent to the study’s focus, the specialized nature of the sample allows us to explore detailed relationships between autistic traits, cognitive abilities, and adaptive behavior. Future studies with larger sample sizes will be essential to confirm these relationships and to explore more nuanced patterns that may not have been detectable in the present study. Additionally, larger studies could allow for more complex subgroup analyses, potentially revealing differences across various subtypes of ASD or in relation to other demographic factors

The primary reason many parents seek formal evaluation and diagnosis for their children is delayed communication and language onset or poor developmental skills compared to peers. This indicates that a large proportion of children with ASD with an early diagnosis struggle with language or have global developmental delays, frequently leading to an IDD diagnosis. Given the young age of our sample and the difficulties in the diagnosis of children with higher-than-average IQ, our sample has an average IQ of 62 (and an upper range of 118) which is not representative of the global autistic population (61). Our sample was underpowered to find weak to medium effects, therefore null effects should be regarded only as a suggestion of the absence of a medium to large (especially for multivariate analyses) effect. Having used a rolling convenience sample, we noticeably missed the opportunity to study specific sub-populations, for instance, the 6A02.1 group, having only 2 children, was excluded by many analyses. Minimum ADOS-2-CS score in our sample was five. ASD diagnosis usually are confirmed starting at a score of four, but given the relation with IQ is frequent that ASD children with high-IQ don’t reach the diagnostic threshold, therefore we could have missed the less obvious end of the spectrum. Age span was also limited to a few years, therefore, generalization to the older population or high-IQ children should be done with extreme caution. Finally, we relied on different instruments given the different ages and ability levels of participants, which may introduce inconsistency among different measures of the same construct.

## Conclusion

With the introduction of DSM-5, different diagnoses were merged into a single spectrum. DSM-5 acknowledges the heterogeneity and requires reporting the presence of IDD, language development, and co-occurring disorders. It also prompts clinicians to indicate the level (ranging from one to three) of support needed for each of the two diagnostic domains. However, after ten years since its introduction, there remains no standardized method for doing so. Levels of support are often used idiosyncratically in clinical practice and left unused in research. Recently, ICD-11 attempted to address this issue by reintroducing a subcategorization based on intellectual and language functioning, resulting in five subcategories. The utility of categories in psychiatry, psychology, or medicine lies in their ability to provide a framework for understanding and classifying complex phenomena. However, for these categories to be truly useful, they should complement continuous traits and variables rather than oversimplify them.

Guided by this principle, we simultaneously examined the effect of continuous and categorical variables in a heterogeneous sample of 56 young autistic children. Our study suggests that while a distinction based on intelligence quotient (IQ) and language functioning could be insightful, the specific distinctions proposed in ICD-11 may not be. A clear distinction between borderline intellectual functioning and normal intelligence is likely necessary. Additionally, reformulating the ICD-11 definition of absence or impairment in functional language to account for developmental effects is crucial. Our findings reveal that when considering the linear effects of IQ variability, autistic characteristics, and the potential influence of co-occurring conditions, three or five primary clusters appear to emerge: (1) Profoundly autistic children with absent functional language (AFL), typically exhibiting an IQ below 50 (and never higher than 80) after the age of six; (2) three, more similar, clusters of children within a moderate range, displaying IQs from mild IDD to BIF, with IQs less than 85, and IFL (group two) or MNIFL (group three), and children under age six with MNIFL but communication skills only mildly impaired (group four); (3) a fifth cluster with normal intelligence and functional language (IQ above 84; MNIFL). We used standard IQ levels in our analyses based on one standard deviation step from the norm, nevertheless, it is not inherently “natural” for biological and social mechanisms to align with the human preference for round numbers. Future research should investigate specific points where clear categorical transitions occur and perform a proper cluster analysis on a larger sample.

Furthermore, while IQ, language, and communication skills significantly impact all adaptive scales, the same holds for autistic traits, even after controlling for IQ in directly assessed adaptive functioning. The multifactorial interplay of autistic traits with other variables makes it practically impossible, and indeed useless, to satisfy the DSM-5 request for assessing the impact of specific ASD domains. Moreover, the high correlation between intelligence and communication skills with an ADOS-2 calibrated score casts shadows on the use of the tool at the extremes of the spectrum, as does the significant leap observed in characteristic behaviors on the PEP-3 in children with an IQ > 85. The ADOS-2 score was calibrated between different modules based on age and language functioning; it should probably be calibrated also for different levels of IQ.

While we observed a strong relationship between parental reports using the VABS-2 and direct assessment with the PEP-3 in communication-related scales, the other two scales from the VABS-2 and PEP-3, once accounting for the correlation created by IQ, autistic traits, and language development, appear largely independent. Therefore, we believe it is essential to use both sources of information for a comprehensive evaluation.

Finally, we noticed an effect of parents’ education level on maladaptive autistic behaviors in children, as well as a negative impact of parents’ age at childbirth on daily living skills. Further research is needed to explore the effects of these variables and inform the development of targeted parent training programs.

This study provides a comprehensive and multidimensional view of adaptive functioning assessment in ASD. It suggests that a fine-tuned classification, combining parent reports, direct assessments, IQ, and diagnosis of autistic traits—despite their high correlation—each contributes uniquely to the overall understanding of autistic children’s needs and strengths. Furthermore, more refined behavioral phenotyping could enhance genetic and biological research by creating meaningful connections across different investigation levels. As we navigate the evolving landscape of ASD, precision remains crucial. Quantitative, objective symptom characterization, beyond mere behavioral observation, is essential. By unraveling the heterogeneity, we move closer to personalized interventions and a deeper understanding of human cognition and experience. We hope this study serves as a starting point for more sophisticated models with larger samples, disentangling the complex relationships among different sources and functions to improve the quality of life for autistic individuals.

## Data Availability

The original contributions presented in the study are included in the article/**Supplementary Material**. Further inquiries can be directed to the corresponding author.
