# Supplementary material for: Autism, intelligence, language, and adaptive behavior, disentangling a complex relationship
Source: Front Psychiatry. 2024 Nov 6;15:1411783. doi: 10.3389/fpsyt.2024.1411783 (PMC11576159; doi:10.3389/fpsyt.2024.1411783)
Supplement: Supplementary file 2 [file DataSheet2.pdf]

Full Results

Correlation Analysis

There was also a moderate correlation between the Age of the child and the mean age of parents $r(56) = .43$, $p < .001$. Year of Education (YoE) of parents correlated with VABS-2-C, $r(56) = .37$, $p = .011$ VABS-2-DL, $r(56) = .28$, $p = .039$, PEP-3-C, $r(56) = .39$, PEP-3-M, $r(56) = .39$, PEP-3-CB, $r(56) = .42$, $p < .003$ for all correlations with PEP-3, strongly correlates with IQ, $r(56) = .43$, $p < .001$, and negative correlation with ADOS-2-CS, $r(56) = -.28$, $p = .039$. Age of Parents was negatively correlated with VABS-2-C, $r(56) = -.32$, $p = .017$, VABS-2-DL, $r(56) = -.47$, $p < .001$, VABS-2-Tot, $r(56) = -.36$, $p = .006$. Substituting the Age of Parents with the Parental Age at Birth (PAB) the correlations were reduced and the only significant remains with VABS-2-DL, $r(56) = -.36$, $p = .006$. Because age, IQ, and ADOS-2 CS were moderately to highly correlated among them and with clinical variables, we re-run the correlation controlling for them. VABS-2 scores remained highly correlated among them, $r(51) = [.51 - .68]$, $p < .001$, but there was no significant correlation with PEP-3 variable, except for PEP-3-CB with VABS-2-Tot, $r(51) = .28$, $p = .044$. PEP-3-CB was also correlated with PEP-3-C, $r(51) = .28$, $p = .047$, and PEP-3-M with PEP-3-C, $r(51) = .61$, $p < .001$. As for parental variables, two correlations survived, the one between YoE and PEP-3-CB, $r(51) = .28$, $p = .044$, and the one between Parental Age / PAB and VABS-2-DL, $r(51) = -.40$, $p = .003$.

Predictors of Parent-Reported Adaptive Functioning

The results of the multivariate tests showed that IQ had a significant effect on the dependent variables, $F(3,49) = 15.7$, $\eta_p^2 = .490$, $p < .001$, as did PAB, $F(3,49) = 6.45$, $\eta_p^2 = .283$, $p < .001$, and YoE, $F(3,49) = 3.84$, $\eta_p^2 = .190$, $p = .015$. Age was marginally significant, $F(3,49) = 2.52$, $\eta_p^2 = .134$, $p = .069$. The between-subjects effects were significant for all dependent variables in the corrected model, VABS-2-C, $F(4,51) = 24.2$, $\eta_p^2 = .655$, $r^2_{adj} = .628$, $p < .001$, VABS-2-DL, $F(4,51) = 17.6$, $\eta_p^2 = .580$, $r^2_{adj} = .547$, $p < .001$, VABS-2-S, $F(4,51) = 11.3$, $\eta_p^2 = .470$, $r^2_{adj} = .428$, $p < .001$. Age had a negative effect on VABS-2-C, $F(1,51) = 4.91$, $\eta_p^2 = .088$, $B = -.221$, $p = .031$, and on VABS-2-DL, $F(1,51) = 4.59$, $\eta_p^2 = .083$, $B = -.202$, $p = .037$, but the multivariate test was not significant. YoE, had a marginal effect on VABS-2-C, $F(1,51) = 3.37$, $\eta_p^2 = .062$, $B = 1.46$, $p = .072$. PAB effect on VABS-2-DL was strong and negative, $F(1,51) = 11.7$, $\eta_p^2 = .187$, $B = -1.28$, $p < .001$, IQ had a strong positive effect on VABS-2-C, $F(1,51) = 44.7$, $\eta_p^2 = .467$, $B = .626$, $p < .001$, VABS-2-DL, $F(1,51) = 23.6$, $\eta_p^2 = .316$, $B = .432$, $p < .001$ and VABS-2-S $F(1,51) = 29.3$, $\eta_p^2 = .365$, $B = .432$, $p < .001$. When ADOS-2-CS was added in covariance, there was no effect of ADOS-2-CS, $F(3,48) = .972$, $\eta_p^2 = .057$, $p = .414$, and the other effects remained similar. Overall η_p^2 increased by only .011 to .018 for different VABS-2 scales. When using ICD-11 groups as a factor, the effect of IQ was reduced, but the pattern of effects remained similar. The same held when using both ICD-11 as a factor and ADOS-2-CS in covariance.

Univariate analysis using VABS-2-Tot instead of the single scales, revealed a strong positive effect of IQ, $F(1,51) = 34.3$, $\eta_p^2 = .402$, $B = .535$, $p < .001$ and a strong negative effect of Age, $F(1, 51) = 14.0$, $\eta_p^2 = .216$, $B = -.347$, $p < .001$. Given the previously found correlation, we added PEP-3-CB in covariate, IQ effect decreased considerably, $F(1, 50) = 2.70$, $\eta_p^2 = .051$, $B = .225$, $p = .107$ and remained a negative effect of Age, $F(1, 50) = 10.5$, $\eta_p^2 = .173$, $B = -.298$, $p = .002$, PEP-3-CB had a strong positive effect, $F(1, 50) = 8.35$, $\eta_p^2 = .143$, $B = .565$, $p = .006$, the same hold when using ADOS-2-CS in covariance. A full analysis is reported as supplementary materials (Sheet S5).

Predictors of Directly Assessed Adaptive Functioning

Multivariate tests, showed a significant effect of IQ, $F(3,49) = 36.1$, $\eta_p^2 = .688$, $p < .001$, and a trend for YoE, $F(3,49) = 1.97$, $\eta_p^2 = .108$, $p = .130$. Between-subjects effects were significant on all dependent variables for the corrected model, PEP-3-C, $F(4,51) = 35.9$, $\eta^2 = .738$, $r^2_{adj} = .718$, $p < .001$, PEP-3-M, $F(4,51) = 19.1$, $\eta^2 = .600$, $r^2_{adj} =$

.568, $p < .001$, PEP-3-CB, $F(4,51) = 35.2$, $\eta^2 = .734$, $r^2_{adj} = .713$, $p < .001$. IQ had a strong positive effect on PEP-3-C, $F(1,51) = 85.8$, $\eta_p^2 = .627$, $B = .558$, $p < .001$, PEP-3-M, $F(1,51) = 42.61$ $\eta_p^2 = .452$, $B = .396$, $p < .001$ and PEP-3-CB, $F(1,51) = 80.4$, $\eta_p^2 = .612$, $B = .549$, $p < .001$; YoE had a marginal, positive effect on all variables PEP-3-C, $F(1,51) = 3.51$, $\eta_p^2 = .064$, $B = .957$, $p = .067$, PEP-3-M, $F(1,51) = 3.10$ $\eta_p^2 = .057$, $B = .910$, $p = .084$ and PEP-3-CB, $F(1,51) = 4.84$, $\eta_p^2 = .087$, $B = 1.14$, $p = .032$. When ADOS-2-CS was added in covariance, there was a high increase in model accuracy. Multivariate tests showed a significant effect of, ADOS-2-CS, $F(3,48) = 12.65$, $\eta_p^2 = .441$, $p < .001$, and IQ, $F(3,48) = 22.6$, $\eta_p^2 = .586$, $p < .001$, and a trend for YoE, $F(3,48) = 2.04$, $\eta_p^2 = .113$, $p = .121$. Between-subjects effects was significant on all dependent variables for the corrected model, PEP-3-C, $F(4,50) = 47.8$, $\eta^2 = .827$, $r^2_{adj} = .810$, $p < .001$, PEP-3-M, $F(4,50) = 19.1$, $\eta^2 = .656$, $r^2_{adj} = .622$, $p < .001$, PEP-3-CB, $F(4,50) = 44.6$, $\eta^2 = .817$, $r^2_{adj} = .799$, $p < .001$. IQ had a strong positive effect on PEP-3-C, $F(1,50) = 45.3$, $\eta_p^2 = .476$, $B = .396$, $p < .001$, PEP-3-M, $F(1,50) = 18.3$ $\eta_p^2 = .268$, $B = .291$, $p < .001$ and PEP-3-CB, $F(1,50) = 41.2$, $\eta_p^2 = .451$, $B = .391$, $p < .001$, and ADOS-2-CS had a strong negative effect on PEP-3-C, $F(1,50) = 25.7$, $\eta_p^2 = .340$, $B = -5.29$, $p < .001$, PEP-3-M, $F(1,50) = 8.17$ $\eta_p^2 = .140$, $B = -3.44$, $p = .006$ and PEP-3-CB, $F(1,50) = 22.6$, $\eta_p^2 = .311$, $B = -5.13$, $p < .001$

Using ICD-11 groups as a factor the effect of IQ was reduced but remained significant, while there was a significant effect of the ICD-11 group. Multivariate tests, showed an effect of IQ, $F(3,44) = 13.0$, $\eta_p^2 = .228$, $p = .009$, and ICD-11, $F(9,138) = 2.89$, $\eta_p^2 = .159$, $p = .004$. Between-subjects effects was significant on all dependent variables for the corrected model, PEP-3-C, $F(7,46) = 24.7$, $\eta^2 = .790$, $r^2_{adj} = .758$, $p < .001$, PEP-3-M, $F(7,46) = 11.2$, $\eta^2 = .631$, $r^2_{adj} = .575$, $p < .001$, PEP-3-CB, $F(7,46) = 30.1$, $\eta^2 = .821$, $r^2_{adj} = .794$, $p < .001$. IQ had a strong positive effect on PEP-3-C, $F(1,46) = 11.1$, $\eta_p^2 = .194$, $B = .357$, $p = .002$, PEP-3-M, $F(1,46) = 5.72$ $\eta_p^2 = .111$, $B = .278$, $p = .021$ and PEP-3-CB, $F(1,46) = 8.02$, $\eta_p^2 = .148$, $B = .286$, $p = .007$. ICD-11 classification had a an effect on PEP-3-C, $F(3,46) = 2.89$, $\eta_p^2 = .159$, $p = .045$, and PEP-3-CB, $F(3,46) = 7.38$, $\eta_p^2 = .325$, $p < .001$, there was no effect on PEP-3-M. Post-hoc pairwise comparisons showed a significant difference between 6A02.0 and all other groups on PEP-3-CB, $MD_{0\text{-}2} = 19.3$, $p < .001$, $MD_{0\text{-}3} = 15.7$, $p = .034$, $MD_{0\text{-}5} = 21.7$, $p = .004$.

When ADOS-2-CS was added in covariance, there was a further increase in model accuracy. Multivariate tests, showed an effect of IQ, $F(3,43) = 3.83$, $\eta_p^2 = .211$, $p = .016$, ICD-11, $F(9,135) = 2.86$, $\eta_p^2 = .161$, $p = .004$ and a strong effect of ADOS-2-CS, $F(3,43) = 11.38$, $\eta_p^2 = .443$, $p < .001$. Between-subjects effects was significant on all dependent variables for the corrected model, PEP-3-C, $F(8,45) = 36.7$, $\eta^2 = .867$, $r^2_{adj} = .843$, $p < .001$, PEP-3-M, $F(8,45) = 11.5$, $\eta^2 = .672$, $r^2_{adj} = .614$, $p < .001$, PEP-3-CB, $F(8,45) = 37.0$, $\eta^2 = .868$, $r^2_{adj} = .845$, $p < .001$. ADOS-2-CS had a strong negative effect on PEP-3-C, $F(1,45) = 26.2$, $\eta_p^2 = .368$, $B = -5.07$, $p < .001$, PEP-3-M, $F(1,45) = 5.70$ $\eta_p^2 = .1112$, $B = -3.03$, $p = .021$, PEP-3-CB, $F(1,45) = 16.\ 0$ $\eta_p^2 = .262$, $B = -4.02$, $p < .001$. IQ had a positive effect on PEP-3-C, $F(1,45) = 8.71$, $\eta_p^2 = .162$, $B = .261$, $p = .005$, PEP-3-M, $F(1,45) = 3.78$ $\eta_p^2 = .078$, $B = .221$, $p = .058$ and PEP-3-CB, $F(1,45) = 5.45$, $\eta_p^2 = .108$, $B = .209$, $p = .024$. ICD-11 classification had a an effect on PEP-3-C, $F(3,45) = 3.42$, $\eta_p^2 = .186$, $p = .025$, and PEP-3-CB, $F(3,45) = 5.91$, $\eta_p^2 = .283$, $p = .002$, there was no effect on PEP-3-M. Post-hoc pairwise comparisons showed a significant difference, on PEP-3-CB, between 6A02.0 and 6A02.2, $MD_{0\text{-}2} = 15.4$, $p = .002$, between 6A02.0 and 6A02.5, $MD_{0\text{-}5} = 17.9$, $p = .008$, while the difference with 6A02.3, was marginal, $MD_{0\text{-}3} = 12.5$, $p = .070$. There was also a difference between 6A02.3 and 6A02.5 on PEP-3-C, $MD_{0\text{-}3} = 9.42$, $p = .036$.

A full analysis is reported as supplementary materials (Sheet S6).

Effect of Intelligence Quotient on Autistic Behaviors

The effect of intelligence quotient on autistic behaviors was assessed using ANOVA, with ADOS-2-CS as a dependent variable. ANOVA was performed using first IQ only and then Age, PAB, and YoE as a further covariate, finally ICD-11 groups were added as factors. The test using PEP-3-CB as a dependent variable is reported in the previous chapter and therefore is not repeated here.

The corrected model using ADOS-2-CS as a dependent variable showed a significant effect of IQ, $F(1,54) = 38.3$, $\eta_p^2 = .415$, $r^2_{adj} = .404$, $p < .001$. When Age, PAB, and YoE were added in covariance, the IQ effect was still significant, F(1,51) = 21.2, $\eta_p^2 = .294$, $p < .001$, $B = -.031$, and there was a trend effect of Age, F(1,51) = 2.82, $\eta_p^2 = .052$, $p = .099$, $B = .012$. Using ICD-11 groups as factors, IQ showed a similar effect, but it was no longer significant, 6A02.0 had a lower ADOS-2-CS score, compared to all other groups, but the difference was not significant.

When PEP-3-C was added in covariation with IQ, Age, PAB and YoE; the effect of IQ was no longer significant, F(1,50) =.348, $\eta_p^2 = .007$, $p = .558$, while there was a large effect of PEP-3-C, F(1,50) = 25.7, $\eta_p^2 = .340$, $p < .001$, $B = -.064$. A full analysis is reported as supplementary materials (Sheet S7).

Effects of Intellectual Development Disorder and Borderline Intellectual Functioning

Instead of using the two levels presented in ICD-11 (Without IDD, IDD), we started with a finer grain analysis using Severe to Moderate ID, IQ ≤ 52, 52 < Mild ≤ 70, 71 < Borderline Intellectual Functioning (BIF) ≤ 85, and Normal Intellectual Functioning (NIF) > 85. Multivariate tests using intellectual functioning groups (IFG) as a factor, PEP-3 scales as dependent variables, and IQ, Age, YoE, and PAB as a covariate, showed a marginally significant effect of IQ, $p = .069$ and IFG, $p = .109$, but the Box's Test of Equality of Covariance Matrices was significant, $p = .010$. A further examination showed a significant between-subject effect of IFG on PEP-3-CB, $p = .032$, but the difference between groups was not significant. Nevertheless, BIF, mild and moderate-severe (MS) IID, led to similar constants of $B_{BIF} = -14.7$, $B_{mild} = -17.7$, and $B_{MS} = -17.6$. Therefore, we re-run the analysis with a separation into only two groups: NIF and BIF or less (BIFL). With only two levels, Box's Test of Equality of Covariance Matrices was no longer significant, and multivariate tests showed an effect of IQ, $F(3,48) = 17.1$ $\eta_p^2 = .517$, $p < .001$, and IFG, $F(3,48) = 4.69$, $\eta_p^2 = .227$, $p = .006$. Between-subjects effects was significant on all dependent variables for the corrected model, PEP-3-C, $F(5,50) = 28.2$, $\eta^2 = .748$, $r^2_{adj} = .712$, $p < .001$, PEP-3-M, $F(5,50) = 15.0$, $\eta^2 = .601$, $r^2_{adj} = .561$, $p < .001$, PEP-3-CB, $F(5,50) = 34.7$, $\eta^2 = .776$, $r^2_{adj} = .754$, $p < .001$. IQ had a strong positive effect on PEP-3-C, $F(1,50) = 50.2$, $\eta_p^2 = .501$, $B = .569$, $p < .001$, PEP-3-M, $F(1,50) = 21.8$, $\eta_p^2 = .303$, $B = .379$, $p < .001$ and PEP-3-CB, $F(1,50) = 28.5$, $\eta_p^2 = .363$, $B = .399$, $p < .001$. IFG had a an effect on PEP-3-CB, $F(1,50) = 9.42$, $\eta_p^2 = .159$, $MD = 15.1$, $p = .003$. Figure 5 shows the different effects of IQ and IFG (two levels) on PEP-3-CB, no other factors were considered for the plot to preserve original data points, without Age, YoE, and PAB, the model parameters were $B_{IQ} = .426$, $p < .001$ and $MD_{IFG} = 17.3$, $p < .001$. No significant effect of IFG was found using VABS-2 scales as dependent variables when IQ was used as a covariate. A full analysis is reported as supplementary materials (Sheet S8).

Effects of Functional Language

For functional language (FL) three levels are present: Absence of Functional Language (AFL), Impaired Functional Language (IFL), Minimal or No Impairment in Functional Language (MNIFL), multivariate tests, using PEP-3 scales as dependent variables, showed an effect of IQ, $F(3,47) = 7.37$ $\eta_p^2 = .320$, $p < .001$, and FL, $F(6,96) = 4.62$, $\eta_p^2 = .224$, $p < .001$. Between-subjects effects was significant on all dependent variables for the corrected model, PEP-3-C, $F(6,49) = 31.5$, $\eta^2 = .794$, $r^2_{adj} = .769$, $p < .001$, PEP-3-M, $F(6,49) = 14.5$, $\eta^2 = .639$, $r^2_{adj} = .595$, $p < .001$, PEP-3-CB, $F(6,49) = 34.3$, $\eta^2 = .808$, $r^2_{adj} = .784$, $p < .001$. IQ had a strong positive effect on PEP-3-C, $F(1,49) = 16.0$, $\eta_p^2 = .246$, $B = .333$, $p < .001$, PEP-3-M, $F(1,49) = 7.11$, $\eta_p^2 = .127$, $B = .241$, $p = .010$ and PEP-3-CB, $F(1,49) = 16.3$, $\eta_p^2 = .249$, $B = .327$, $p < .001$. LF had a an effect on PEP-3-C, $F(2,49) = 6.66$, $\eta_p^2 = .214$, $p = .003$, a marginal effect on PEP-3-M, $F(2,49) = 2.66$, $\eta_p^2 = .098$, $p = .080$ and strong effect on PEP-3-CB, $F(2,49) = 9.36$, $\eta_p^2 = .276$, $p < .001$. Post-hoc pairwise comparisons showed a significant difference between AFL and the two other groups on PEP-3-C, $MD_{AFL\text{-}IFL} = -11.0$, $p = .009$ and $MD_{AFL\text{-}MNIFL} = -17.1$, $p = .004$, and a significant difference

between MINLF and the two other groups on PEP-3-CB, $MD_{MNIFL-IFL}$ = 15.3, $p < .001$ and $MD_{MNIFL-AFL}$ = 18.7, p = .001. Using VABS-2 scales as dependent variables when IQ or IQ and ADOS-2-CS were in covariate, while without IQ and ADOS-2-CS, the multivariate effect of FL was significant, $F(6,98) = 4.62$, $\eta_p^2 = .220$, $p < .001$ as all the Between-subjects effects.

In a second ANOVA analysis, we studied the effect of FL on IQ. Between-subjects effects were significant for the corrected model, $F(6,49) = 18.7$, $\eta^2 = .696$, $r^2_{adj} = .658$, $p < .001$. Age and ADOS-2-CS had small negative effect, $F(1,49) = 3.83$, $\eta_p^2 = .072$, $B = -.192$, $p = .056$, ADOS-2-CS, $F(1,49) = 3.91$, $\eta_p^2 = .074$, $B = -3.73$, $p = .054$. The effect of FL was very large, $F(2,49) = 19.1$, $\eta_p^2 = .439$, $p < .001$ and there was a significant difference ($p < .001$) among all three groups, after accounting for demographic variables and ADOS-2-CS, $M_{AFL} = 46.2$, $M_{IFL} = 67.5$ and M_{MNIFL} = 84.1.

Finally, we also introduced PEP-3-C in covariance. There was no ADOS-2-CS effect on IQ, but there was a large effect of PEP-3-C, $F(1,48) = 11.2$, $B = .804$, $\eta_p^2 = .189$, $p = .002$, and the effect of FL was still present, $F(2,48) = 3.64$, $\eta_p^2 = .132$, $p = .034$ with estimated parameters: $B_{AFL} = -20.7$, $p = .010$; $B_{IFL} = -11.9$, $p = .042$ and $MD_{AFL-MNIFL} = -20.7$, $p = .031$.

A post-hoc analysis, the reason for which will be explained in the discussion, was performed to study internal differences of the AFL group with a subdivision based on Age Group (AG) between younger (AFL-Y; Age < 72 months) and older children (AFL-O), this post-hoc analysis also included participants classified as 6A02.Z (a second analysis without them led to similar results). There was a strong association among all adaptive and diagnostic variables (except VABS-2-S which was trending but non-significant) and group membership, with a higher score (lower for ADOS-2-CS) in AFL-Y. AFL-Y had an average age of 41 months while AFL-O was 82 months, there was no difference in YoE or PAB but IQ was 58.8 in AFL-Y and 40.8 in AFL-O, $p = .009$; PEP-3-C was 92.8 in AFL-Y and 77.4 in AFL-O, $p = .001$; PEP-3-CB was 91.3 in AFL-Y and 78.5 in AFL-O, $p = .004$; VABS-2-C was 54.7 in AFL-Y and 39.4 in AFL-O, $p = .036$; VABS-2-Tot was 65.3 in AFL-Y and 40.2 in AFL-O, $p = .001$. Furthermore, also the three subscales of PEP-3-C (preverbal/verbal IQ, expressive and receptive language) were significantly different, $p < .019$. A full analysis is reported as supplementary materials (Sheet S9).

Predictive Value of Parental Assessment of Adaptive Functioning on Direct Assessment

PEP-3 scales were used as dependent variables, while VABS-2 scales were used as covariates.

Multivariate tests showed a significant effect of VABS-2-C, $F(3,50) = 10.9$, $\eta_p^2 = .396$, $p < .001$. Between-subjects effects were significant on all dependent variables for the corrected model, PEP-3-C, $F(3,52) = 32.3$, $\eta^2 = .651$, $r^2_{adj} = .630$, $p < .001$, PEP-3-M, $F(3,52) = 17.0$, $\eta^2 = .495$, $r^2_{adj} = .466$, $p < .001$, PEP-3-CB, $F(3,52) = 27.1$, $\eta^2 = .610$, r^2_{adj} = .587, $p < .001$. VABS-2-C had a strong positive effect on PEP-3-C, $F(1,52) = 33.0$, $\eta_p^2 = .388$, $B = .604$, $p < .001$, PEP-3-M, $F(1,52) = 13.7$ $\eta_p^2 = .209$, $B = .383$, $p < .001$ and PEP-3-CB, $F(1,52) = 16.8$, $\eta_p^2 = .245$, $B = .459$, $p < .001$.

Adding IQ, Age, YoE, and PAB in covariate, VABS-2-C was still significant, $F(3,46) = 3.39$, $\eta_p^2 = .181$, $p = .026$, and also IQ had a significant effect, $F(3,46) = 11.8$, $\eta_p^2 = .435$, $p < .001$,. Between-subjects effects were significant on all dependent variables for the corrected model, PEP-3-C, $F(7,48) = 25.1$, $\eta^2 = .785$, $r^2_{adj} = .754$, $p < .001$, PEP-3-M, $F(7,48) = 11.7$, $\eta^2 = .620$, $r^2_{adj} = .564$, $p < .001$, PEP-3-CB, $F(7,48) = 22.3$, $\eta^2 = .765$, $r^2_{adj} = .731$, $p < .001$. VABS-2-C had a strong positive effect on PEP-3-C, $F(1,48) = 9.71$, $\eta_p^2 = .168$, $B = .323$, $p = .003$, while IQ had a strong effect on, PEP-3-C, $F(1,48) = 27.7$, $\eta_p^2 = .366$, $B = .415$, $p < .001$, PEP-3-M, $F(1,48) = 12.7$ $\eta_p^2 = .210$, $B = .307$, $p < .001$ and PEP-3-CB, $F(1,48) = 25.4$, $\eta_p^2 = .346$, $B = .418$, $p < .001$.

Adding LF as a factor, VABS-2-C became barely significant, $F(3,44) = 2.49$, $\eta_p^2 = .145$, $p = .072$, IQ had still a significant effect, $F(3,44) = 4.00$, $\eta_p^2 = .214$, $p = .013$, and there was a significant effect of LF, $F(6,90) = 3.70$, η_p^2 =

.198 p = .002. Between-subjects effects was significant on all dependent variables for the corrected model, PEP-3-C, $F(9,46) = 23.7$, $\eta^2 = .823$, $r^2_{adj} = .788$, $p < .001$, PEP-3-M, $F(9,46) = 9.41$, $\eta^2 = .648$, $r^2_{adj} = .579$, $p < .001$, PEP-3-CB, $F(9,46) = 23.9$, $\eta^2 = .824$, $r^2_{adj} = .790$, $p < .001$. VABS-2-C had a positive effect on PEP-3-C, $F(1,46) = 7.00$, $\eta_p^2 = .132$, $B = .260$, $p = .011$, while IQ had an effect on, PEP-3-C, $F(1,46) = 8.49$, $\eta_p^2 = .156$, $B = .259$, $p = .005$, PEP-3-M, $F(1,46)$ = 3.75 $\eta_p^2 = .075$, $B = .199$, $p = .059$ and PEP-3-CB, $F(1,55) = 8.69$, $\eta_p^2 = .159$, $B = .263$, $p = .008$. Furthermore, LF had a significant effect on PEP-3-C, $F(2,46) = 4.87$, $\eta_p^2 = .175$, $p = .012$ and PEP-3-CB, $F(2,46) = 7.74$, $\eta_p^2 = .252$, $p = .001$. Post-hoc pairwise comparisons showed a significant difference between AFL and the two other groups on PEP-3-C, $MD_{AFL-IFL} = -9.49$, $p = .026$ and $MD_{AFL-MNIFL} = -14.3$, $p = .017$, and a significant difference between MINLF and the two other groups on PEP-3-CB, $MD_{MNIFL-IFL} = 14.4$, $p = .002$ and $MD_{MNIFL-AFL} = 17.4$, $p = .003$. Adding ADOS-2-CS as a covariate, the multivariate effect of VABS-2-C was no longer significant, $p = .090$, but the univariate effect on PEP-3-C was still significant, $p = .016$. The pattern of all other effects was similar and ADOS-2-CS. Between-subjects effects was significant on all dependent variables for the corrected model, PEP-3-C, $F(10,45) = 34.1$, $\eta^2 = .883$, $r^2_{adj} = .857$, $p < .001$, PEP-3-M, $F(10,45) = 9.67$, $\eta^2 = .682$, $r^2_{adj} = .612$, $p < .001$, PEP-3-CB, $F(10,45) = 29.6$, $\eta^2 = .868$, $r^2_{adj} = .839$, $p < .001$; ADOS-2-CS effect was on all dependent variables, PEP-3-C, $F(1,45)$ = 23.4, $\eta_p^2 = .883$, $p < .001$, PEP-3-M, $F(1,45) = 4.87$, $\eta_p^2 = .682$, $p = .032$, PEP-3-CB, $F(1,45) = 15.0$, $\eta_p^2 = .868$, $p < .001$.

A full analysis is reported as supplementary materials (Sheet S10).

To assess the predictive value of the total VABS-2 scale (comprising also motor skills for children younger than six), PEP-3 scales were used as dependent variables, while VABS-2-Tot was used as a covariate.

Multivariate tests showed a significant effect of VABS-2-Tot, $F(3,52) = 24.5$, $\eta_p^2 = .585$, $p < .001$. Between-subjects effects were significant on all dependent variables for the model, PEP-3-C, $F(1,54) = 59.3$, $\eta^2 = .523$, $r^2_{adj} = .514$, $p < .001$, PEP-3-M, $F(1,54) = 35.9$, $\eta^2 = .399$, $r^2_{adj} = .388$, $p < .001$, PEP-3-CB, $F(1,54) = 68.9$, $\eta^2 = .561$, $r^2_{adj} = .553$, $p < .001$.

Adding IQ, Age, YoE, and PAB in covariate, VABS-2-Tot was still significant, $F(3,48) = 3.02$, $\eta_p^2 = .159$, $p = .039$, and IQ had a strong significant effect, $F(3,48) = 15.4$, $\eta_p^2 = .491$, $p < .001$, Between-subjects effects was significant on all dependent variables for the corrected model, PEP-3-C, $F(5,50) = 31.5$, $\eta^2 = .759$, $r^2_{adj} = .735$, $p < .001$, PEP-3-M, $F(5,50) = 15.6$, $\eta^2 = .610$, $r^2_{adj} = .571$, $p < .001$, PEP-3-CB, $F(5,50) = 33.9$, $\eta^2 = .772$, $r^2_{adj} = .749$, $p < .001$. VABS-2-Tot had a positive effect on PEP-3-C, $F(1,50) = 4.37$, $\eta_p^2 = .080$, $p = .042$, and PEP-3-CB, $F(1,50) = 8.35$, $\eta_p^2 = .143$, $p = .006$, while IQ had a strong positive effect on all variables. Multivariate test for YoE was not significant but "trending", with $F(3,48) = 2.04$, $\eta_p^2 = .113$, $p = .121$, the effect was positive on all variables, PEP-3-C, $F(1,50)$ = 3.40, $\eta_p^2 = .064$, $B = .914$, $p = .071$, PEP-3-M, $F(1,50) = 2.95$, $\eta_p^2 = .056$, $B = .886$, $p = .092$, PEP-3-CB, $F(1,50) = 4.97$, $\eta_p^2 = .090$, $B = 1.08$, $p = .030$.

Adding LF as a factor, and the ADOS-2-CS as a covariate, the VABS-2-Tot effect was no longer significant, but the direction and magnitude of the VABS-2-Tot, IQ, and YoE effect were similar. The effects of LF and ADOS-2-CS were similar to the ones for the previously reported VABS-2 subscales model.

A full analysis is reported as supplementary materials (Sheet S11).
